# Proteome-wide analysis of chaperone-mediated autophagy targeting motifs

**DOI:** 10.1371/journal.pbio.3000301

**Published:** 2019-05-31

**Authors:** Philipp Kirchner, Mathieu Bourdenx, Julio Madrigal-Matute, Simoni Tiano, Antonio Diaz, Boris A. Bartholdy, Britta Will, Ana Maria Cuervo

**Affiliations:** 1 Department of Developmental and Molecular Biology, Albert Einstein College of Medicine, Bronx, New York, United States of America; 2 Institute for Aging Studies, Albert Einstein College of Medicine, Bronx, New York, United States of America; 3 Department of Cell Biology, Albert Einstein College of Medicine, Bronx, New York, United States of America; 4 Department of Medicine, Albert Einstein College of Medicine, Bronx, New York, United States of America; Institute of Basic Medical Sciences, NORWAY

## Abstract

Chaperone-mediated autophagy (CMA) contributes to the lysosomal degradation of a selective subset of proteins. Selectivity lies in the chaperone heat shock cognate 71 kDa protein (HSC70) recognizing a pentapeptide motif (KFERQ-like motif) in the protein sequence essential for subsequent targeting and degradation of CMA substrates in lysosomes. Interest in CMA is growing due to its recently identified regulatory roles in metabolism, differentiation, cell cycle, and its malfunctioning in aging and conditions such as cancer, neurodegeneration, or diabetes. Identification of the subset of the proteome amenable to CMA degradation could further expand our understanding of the pathophysiological relevance of this form of autophagy. To that effect, we have performed an in silico screen for KFERQ-like motifs across proteomes of several species. We have found that KFERQ-like motifs are more frequently located in solvent-exposed regions of proteins, and that the position of acidic and hydrophobic residues in the motif plays the most important role in motif construction. Cross-species comparison of proteomes revealed higher motif conservation in CMA-proficient species. The tools developed in this work have also allowed us to analyze the enrichment of motif-containing proteins in biological processes on an unprecedented scale and discover a previously unknown association between the type and combination of KFERQ-like motifs in proteins and their participation in specific biological processes. To facilitate further analysis by the scientific community, we have developed a free web-based resource (KFERQ finder) for direct identification of KFERQ-like motifs in any protein sequence. This resource will contribute to accelerating understanding of the physiological relevance of CMA.

## Abstract 2

Cells use a sophisticated code to sort proteins that must be retained for reuse from those that need to be sent to lysosomes for degradation and recycling. These authors develop tools to identify the selective lysosomal degradation motifs and use them to start breaking this code.

## Introduction

Autophagy is an essential cellular pathway involved in homeostasis maintenance through degradation and recycling of almost every cellular component, from proteins to lipids and organelles in lysosomes [1]. Three main types of autophagy coexist in most mammalian cells: macroautophagy, chaperone-mediated autophagy (CMA), and microautophagy [1]. All three share the lysosome as the final catabolic compartment, but their mechanisms for cargo delivery and regulation differ considerably.

This work focuses on CMA, which degrades single proteins that carry a specific combination of five amino acids, named “KFERQ-motif” after the first of such pentapeptides was discovered by the late J.F. Dice near the N terminus of ribonuclease A [2]. This motif is used by the chaperone heat shock cognate 71 kDa protein (HSC70) to bind the substrate protein and direct it to the lysosomal membrane. Here, the substrate/HSC70 complex binds to the essential receptor for CMA, the lysosome-associated membrane protein type 2A (LAMP-2A). Binding promotes LAMP-2A multimerization into a higher-molecular–order complex at the membrane, where, after unfolding, the substrate protein is translocated into the lysosome [3, 4]. A lysosomal resident HSC70 isoform assists with translocation of the substrate protein across the membrane towards the lysosomal lumen, where it is degraded by the acidic hydrolases.

CMA is ubiquitously detectable at basal levels in almost all mammalian cells experimentally tested so far [5]. CMA activity increases in response to stressors such as starvation [6], lipotoxicity [7], proteotoxicity [8], hypoxia [9], oxidative stress [10], or DNA damage [11]. The high selectivity of CMA confers this pathway a pivotal role in the fine-tuning of a variety of processes, including T-cell activation, DNA repair, cell cycle regulation, glucose and lipid catabolism, cell growth, or cell survival programs [4, 11–13]. Interestingly, CMA activity decreases with age [14] and upon metabolic challenges like high-lipid diets [7]. CMA is also a target of the toxicity of pathogenic proteins involved in degenerative processes, thus linking CMA dysfunction to conditions such as neurodegenerative diseases [8, 15], cancer [16], or metabolic diseases including diabetic nephropathy [17] and fatty liver [18].

**Fig 1:**
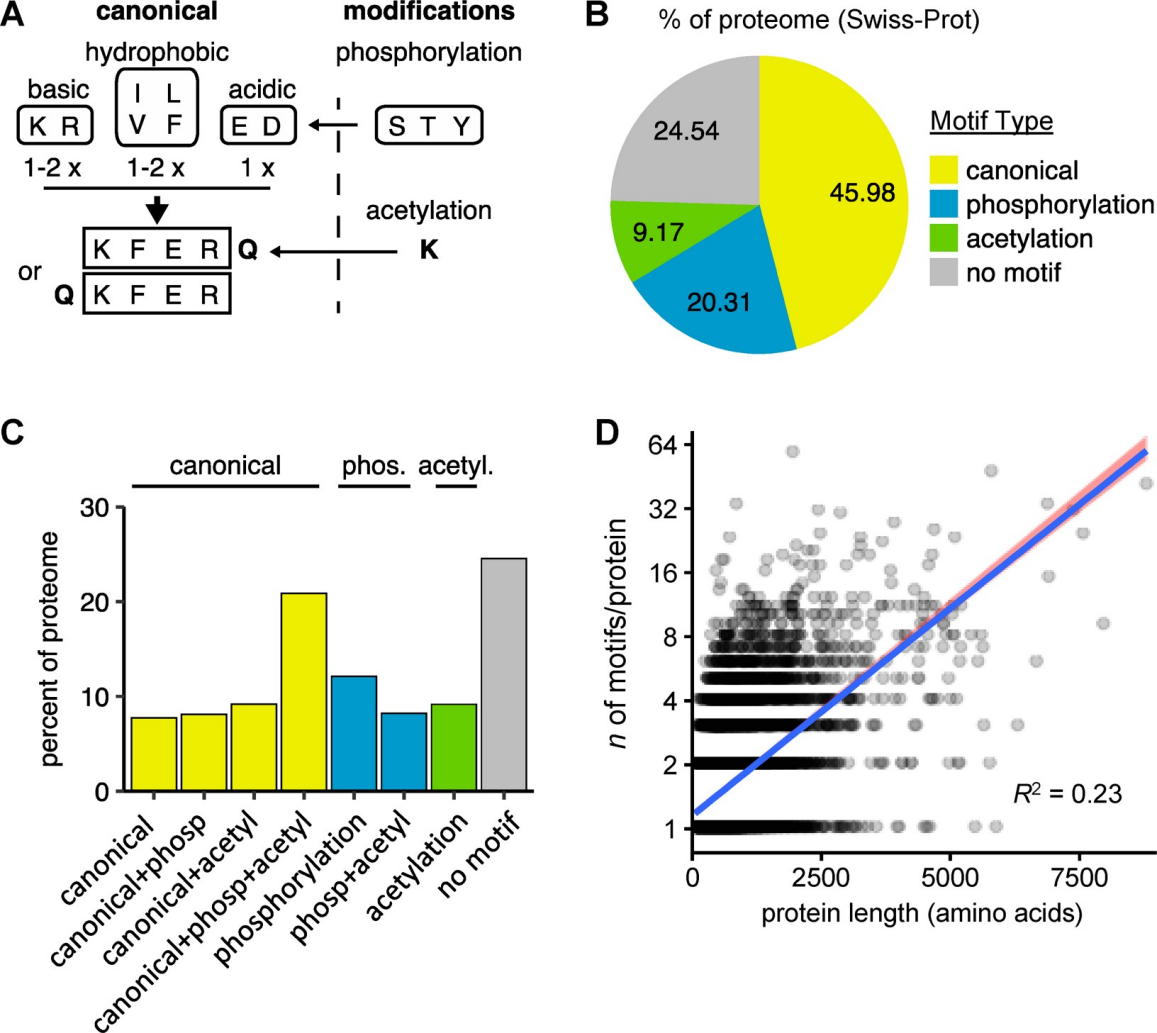
Frequency and types of KFERQ-like motifs in the human proteome. (**A**) Scheme of the building rules of canonical, phosphorylation-, and acetylation-generated KFERQ-like motifs. (**B**) Percentage of proteins in the human proteome (filtered for reviewed entries) harboring the indicated classes of KFERQ-like motifs. Occurrence of motifs is ranked as canonical > phosphorylation-generated > acetylation-generated, and proteins with a combination of motifs are assigned to a group based on their highest-ranking motif. (**C**) Percentages of the reviewed human proteome harboring particular combinations of KFERQ-like motifs (generated by splitting the data from Fig 1B into all possible motif combinations). (**D**) Linear model for the correlation between the number of canonical motifs and protein length. The blue line represents the ordinary least squares regression with 95% confidence intervals (red area) using the relationship: log_2_(number of motifs) = protein length. Three very long proteins were removed as outliers (Cook’s distance > 1). *R*^2^ is the goodness-of-fit statistic for the fitted model. acetyl., acetylation; phosp, phosphorylation.

Specificity in substrate selection by CMA is attained through the special recognition mechanism between the substrate proteins and HSC70 [2]. This interaction requires the presence of the KFERQ-like motif in the amino acid sequence of the substrate protein. Seminal studies using ribonuclease A demonstrated that this motif is necessary and sufficient for protein degradation by CMA. Mutating the pentapeptide abolished lysosomal catabolism of ribonuclease A [2], whereas inserting the first 11 amino acids of ribonuclease A into non-CMA substrate proteins targeted them for lysosomal degradation. Indeed, the latter manipulation is the basis for a fluorescent reporter system developed to measure CMA activity [5]. Phage display binding assays using HSC70 to identify and refine the motif revealed that the specific amino acids are not important but that the affinity of binding is determined by their physical properties. Thus, a canonical KFERQ-like motif must contain the following (**Fig 1A**): (i) one or two of the positively charged residues: K, R; (ii) one or two of the hydrophobic residues: I, L, V, F; (iii) one of the negatively charged residues: D, E; and (iv) one glutamine (Q) on either side of the pentapeptide [19]. As a result, several different combinations of amino acids can result in a KFERQ-like motif within the sequence of a protein. Furthermore, posttranslational modifications (PTMs) such as phosphorylation [20] or acetylation [21] can create a KFERQ-like (canonical) motif from a putative motif. Phosphorylated amino acids can substitute for acidic ones and an acetylated lysine can take the place of the glutamine, thus increasing the flexibility and degree of modulation available to CMA substrates (**Fig 1A**).

Affinity antibodies against the KFERQ pentapeptide were able to precipitate up to 35% of the cytosolic proteins in fibroblasts, leading to the conclusion that this may be the approximate number of proteins potentially amenable to be degraded by CMA [22]. However, for a motif to be used for degradation, it needs to be accessible for HSC70. Certain proteins do not have an exposed motif under regular circumstances, but conditions that promote protein disassembly, such as oxidative stress, make this motif accessible for HSC70 binding and subsequent degradation [10]. Likewise, protein cleavage, protein disassembly from the protein complex, or release of membrane-bound proteins may expose previously buried motifs.

Recent work has shown that binding of HSC70 to KFERQ-like motifs can also target substrates for degradation through endosomal microautophagy (eMI), in which substrate proteins are sequestered in intraluminal vesicles budding into the lumen of multivesicular endosomes [23]. In contrast to CMA, the KFERQ-like motif is necessary but not sufficient for eMI targeting in mammals [23].

The fact that potentially more than one third of the cytosolic proteins carry a KFERQ-like motif makes CMA an important mechanism for regulation of cellular proteostasis and cell adaptability to challenges. Given the growing interest in the pathophysiological role of CMA in humans, a reliable method to determine the presence of these motifs is needed to assist in the identification of potential CMA substrates. The lack of such a resource, combined with misconceptions about the motif architecture and its modifications, has motivated us in this work to perform a proteome-wide in silico search for KFERQ-like motifs in the human proteome and determine their abundance, location, and preferred amino acid composition. Furthermore, we have investigated the evolutionary conservation of motifs in the context of species able and unable to perform CMA. Lastly, we have performed an enrichment analysis of proteins harboring particular classes of KFERQ-like motifs in biological processes.

Our work offers unprecedented insight into KFERQ-like motifs that should help to gain a better understanding of CMA. We also provide to the scientific community interested in CMA a free web-based resource to search for KFERQ-like motifs in single proteins, protein batches, or full proteomes.

## Results

### Frequency of KFERQ-like motifs in the human proteome

Using the KFERQ-like motif construction rules experimentally defined originally [2] (**Fig 1A**), we analyzed all sequences of the human UniProt reference proteome (uniprot.org, UP000005640, 70,952 proteins) for the presence of KFERQ-like motifs. The analysis was then restricted to reviewed Swiss-Prot entries (20,165 proteins), excluding proteins transcribed from open reading frames without direct experimental confirmation (TrEMBL). This filtering step strongly reduced the number of short proteins (<250 amino acids, **S1A Fig**) that are often protein fragments.

It is not unusual that proteins contain more than one KFERQ-like motif. However, because it has been experimentally demonstrated that increasing the number of motifs in a protein does not accelerate its rate of degradation by CMA [19], we imposed a hierarchy on the types of motifs for protein grouping purposes. Canonical motifs are those already present in the unmodified protein sequence and are the best characterized. Phosphorylation- and acetylation-generated motifs (putative) are less well described and require PTMs in the protein sequence. Consequently, we categorized the results into four groups: (i) proteins containing at least one canonical motif, without factoring in the presence of putative motifs (canonical); (ii) proteins without canonical motifs but containing at least one phosphorylation-generated motif (phosphorylation-generated); (iii) proteins with only acetylation-generated motifs; and (iv) proteins without any motif. Although in specific circumstances the flanking Q residue in a motif can be replaced by asparagine (N) [24], due to the current lack of experimental information on the permissibility of this replacement, we did not include those motifs in our analysis (for more details, see the Discussion section). In the restricted data set, 45.98% of sequences contained at least one canonical motif, 20.31% contained no canonical motif but a phosphorylation-generated, and 9.17% contained only acetylation-generated motifs (**Fig 1B**). A similar overall motif distribution was found in the complete UniProt human proteome, including unreviewed TrEMBL entries, although the percentage of proteins without motif was higher (**S1B Fig**).

We next analyzed, within each group, the occurrence of additional motifs and found that, for proteins bearing at least one canonical motif, about 20% also contain phosphorylation-generated motifs, 20% acetylation-generated motifs, and almost half of them (46.5%) contain all three (**Fig 1C**). In the case of proteins with only putative motifs, one third contained both phosphorylation- and acetylation-generated motifs (**Fig 1C**). Analysis of the number of motifs of each type in proteins grouped according to **Fig 1B** revealed that the most common occurrence was for proteins to carry only one motif, independently of the motif type (**S1C Fig**). This indicates that when proteins contain motifs of different classes, most of the time, each class is only represented once. To determine whether the presence of more than one motif was more common in longer proteins, we analyzed a possible correlation between protein length and number of canonical motifs (**Fig 1D**). The low correlation coefficient (*R*^2^ = 0.234) indicates that protein length is a poor predictor for the number of motifs per protein.

### Position of motifs within proteins

**Fig 2:**
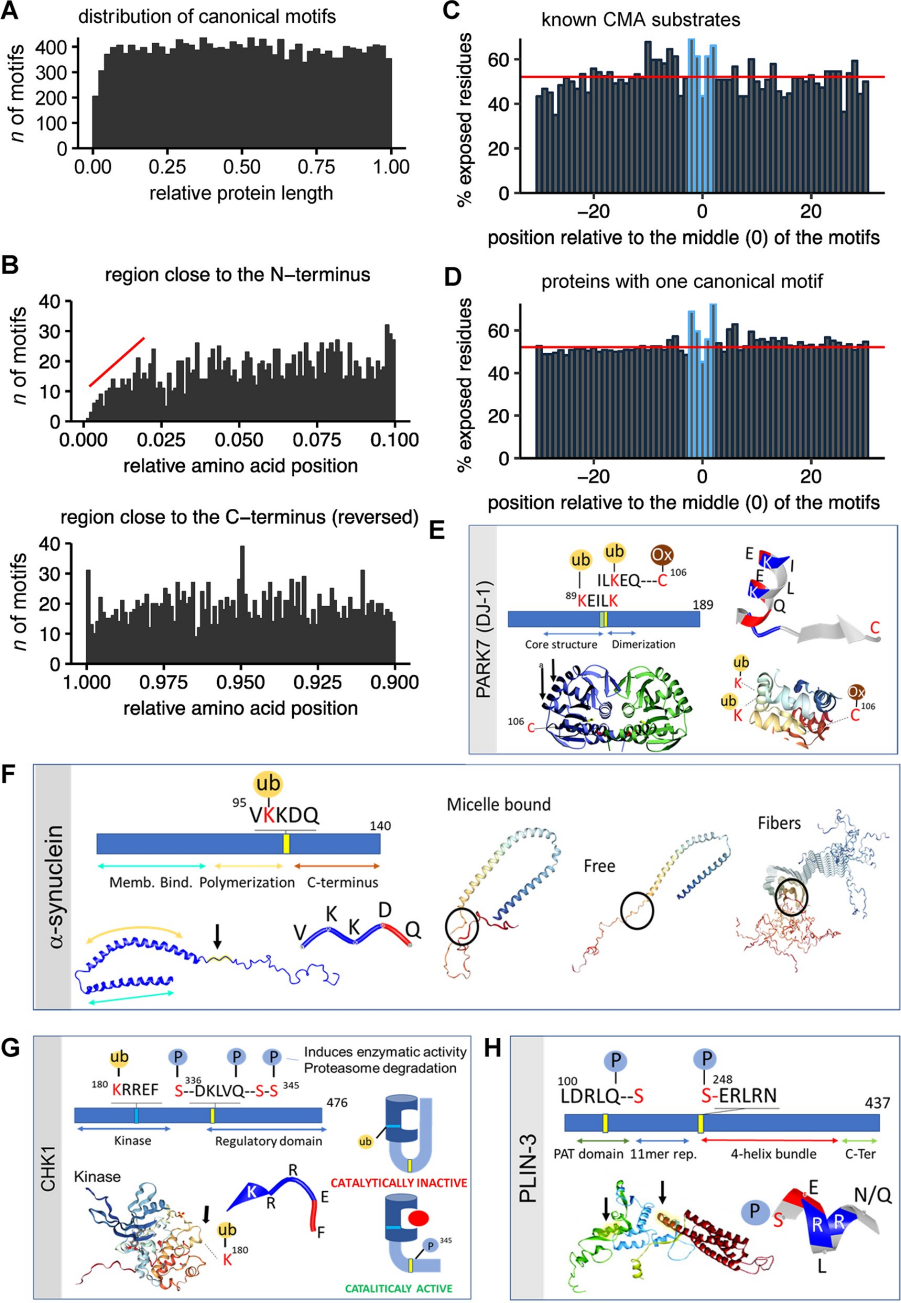
Distribution of KFERQ-like motifs within protein sequences. (**A**) Distribution of canonical KFERQ-like motifs along the protein length (normalized to a scale from 0 [N-terminus] to 1 [C-terminus]). The histograms show the count of motifs at the relative position with a bin size of 0.02. (**B**) The first 10% (N-terminus; top) and last 10% (C-terminus; bottom) of the normalized protein length in Fig 2A, shown here with a bin size of 0.001. The C-terminal plot (bottom) is mirrored for easier comparison. The red line indicates the slope of the reduction in KFERQ-like motifs. (**C, D**) Bar plots showing the average of exposed amino acids, as predicted from the primary sequence, using JPred4 for proteins validated as CMA substrates (**C**) or proteins in the human proteome harboring one canonical motif (**D**). For each protein, a region ±30 amino acids around the central amino acid of the motifs was isolated and aligned on the KFERQ-like motifs. The percentage of exposed residues was then calculated for each position. The red line indicates the mean percentage of exposure for all amino acids in all investigated proteins. Amino acids that are part of the KFERQ-like motifs are highlighted in blue. (**E-H**) Examples of domain localization and experimentally confirmed PTMs in KFERQ-like motifs of DJ-1 (**E**), alpha-synuclein (**F**), CHK1 (**G**) and PLIN3 (**H**). Canonical motifs are marked as yellow bars, phosphorylation-generated in blue, and acetylation-generated in green. Protein structures were obtained from the RCSBPDB protein data bank (rcsb.org) using PBD IDs 1j42 (for DJ-1 [25]); 1XQ8, 2KKW, and 2N0A (for alpha-synuclein [26]); 4FSM (for Chk1 [27]); and 1SZI (for PLIN3 [28]). The structures of the KFERQ-like motifs are shown as strings and ribbons colored based on amino acid properties. PTMs shown: ubiquitylation (ub), phosphorylation (P), and oxidation (Ox). Arrows: location of the motif in the protein structure. The cartoon in (G) depicts the conformational change in Chk1 that releases autoinhibition of its catalytic activity. CHK1, checkpoint kinase 1; CMA, chaperone-mediated autophagy; Memb. Bind., Membrane Binding; Ox, oxidation; P, phosphorylation; PARK7, Parkinsonism associated deglycase; PAT, perilipin/ADRP/TIP47; PLIN3, perilipin 3; PTM, posttranslational modification; ub, ubiquitylation.

The number of experimentally validated CMA substrate proteins (<50) is still too low to identify possible preferences in the position of the KFERQ-like motifs within the protein sequence. **S2A and S2B Fig** shows the absolute and relative position of motifs in a subset of 16 of the validated CMA substrates (summarized in [4]). However, taking advantage of our proteome-wide search for these motifs, we analyzed the distribution of canonical and putative motifs along the protein length. We anticipated that only very strong position preferences might become evident because the tested proteins have highly variable sizes, domain structure, and function. For the group of proteins with canonical KFERQ-like motifs, we observed a largely uniform motif distribution along the protein length (**Fig 2A**). Interestingly, we also noticed a decrease in the number of motifs close to the N-terminus of proteins compared with the C-terminus (**Fig 2B**). This difference is not due to the presence of an initiator methionine, because it was still noticeable even when N-terminal methionine residues were not counted towards protein length (**S2C Fig**). Similar results were obtained for the putative phosphorylation- and acetylation-generated motifs that were equally distributed along the protein length, with a decrease in frequency close to the N-terminus of proteins compared with the C-terminus (**S2D Fig**). The mean number of canonical motifs in the first 2.5% of the protein length was 42% lower than in the remaining 97.5% of the protein. This reduction was 35% for phosphorylation-generated and 43% for acetylation-generated motifs, respectively. Future studies on the preference for the C-terminal region may shed new light on the mechanism of substrate recognition by HSC70 or on the dynamics of the chaperone/substrate complex once reaching the lysosomal membrane.

We also considered the location of the motif in the fully folded proteins, because for HSC70 binding, the motif should be accessible at the protein surface. We followed the approach used to study the relation between protein structure and motif location for other small linear motifs (SLiMs) [29] that share characteristics with KFERQ-like motifs, such as their short lengths (average of six amino acids) and degenerated sequences [30]. To estimate the accessibility of a motif, we predicted the solvent accessibility of the amino acids in the motif and the surrounding protein region with the JPred4 algorithm [31]. Using four experimentally confirmed CMA substrates, we validated that the classification of residues into buried and exposed predicted by the JPred4 algorithm closely follows the classification obtained from their protein crystal structures (**S2E Fig**). We next investigated the motifs and their surrounding regions in 24 experimentally confirmed CMA substrates (**S1 Table**) using the predicted solvent accessibility. **Fig 2C** shows the average percentage of exposed amino acids in KFERQ-like motifs (marked in blue) with a flanking region of ±30 amino acids around the central motif position. The mean percentage of exposed residues over all investigated proteins (red line) is close to 50%, in line with an equal probability for an amino acid to be classified as exposed or buried. Interestingly, the amino acids in the motif and in close proximity upstream of the motif (approximately 8 residues) are more frequently classified as exposed. This is especially striking for the flanking amino acids inside the motif, while the central amino acid is less exposed (**Fig 2C**). Very similar accessibility properties in the residues at the motif were observed when we performed the same type of analysis in >1,000 proteins in the human proteome bearing one canonical motif (**Fig 2D**). In this case, while the solvent exposure of amino acids outside of the KFERQ-like motif was closer to the baseline distribution of 50%, the flanking amino acids that form part of the motif were still clearly more frequently exposed and the central amino acid was again more buried (**Fig 2D**). These results support a critical function to the flanking amino acids of the motif, in agreement with the experimental observation that mutation of the flanking Q and the amino acid next to it is sufficient to disrupt HSC70 binding [8, 11–13, 15].

Partial protein unfolding, often associated with protein damage or aberrant synthesis, might make KFERQ-like motifs accessible to HSC70 for targeting to CMA as part of its role in protein quality control. However, recent studies support that timely removal of still functional proteins by CMA is behind the ability of this autophagic pathway to regulate multiple intracellular processes (i.e., glycolysis, lipolysis, cell cycle arrest, etc.) [11, 18]. In those instances, because HSC70 recognizes the motif in the fully folded protein, it is likely that location in specific protein domains and/or PTMs in the motifs and in nearby areas contribute to modulating HSC70 binding. To start gaining insights into this regulated substrate recognition, we used 24 experimentally validated substrates (72 motifs total) and evaluated the protein domains where motifs localize and their possible PTMs (**S2 Table**). Despite the small sample size, we found that close to 40% of motifs were in protein domains known to mediate protein–protein interactions (**S2 Table**). We also identified motifs in regions described to be important for protein structure (27%) or protein activity (31%). Analysis of experimentally validated PTMs—which can generate or disrupt existing motifs—revealed abundance of ubiquitylation and acetylation events in the KFERQ-motifs of this subset of CMA substrates (**S2 Table**). Other modifications, such as sumoylation, methylation, succinylation, and neddylation, that will disrupt recognition of the motif by HSC70 were also identified (**S2 Table**). **Fig 2E–2H** shows a series of vignettes illustrating how the information on location and PTMs of KFERQ-like motifs could be used to infer their possible impact on regulated degradation of a protein. For example, in the case of the transcriptional regulator DJ-1 (PARK7 locus) (**Fig 2E**) the overlapping acetylation-generated motif and the canonical motif are in an alpha helix of the core structure region, 10 residues upstream of the cysteine (^106^C) shown to be key for activation of DJ-1 by free radicals [32]. It is possible that the oxidative status of ^106^C changes the exposure of the two K residues in the motif to promote HSC70 binding, or to prevent it through their already described ubiquitylation (^89^K [33] or ^93^K [34]). The only canonical motif present in α-synuclein, a protein tightly related with Parkinson disease pathology, is right at the transitional region between the α-helix structured polymerization region and the disorganized C terminus (**Fig 2F**). Upon analysis of the position of this motif in currently available structures of this protein, we noticed that association of α-synuclein to membranes (as part of its physiological function [35]) buries the KFERQ-like motif (**Fig 2F**). Membrane binding masking the KFERQ-like motif may prevent the degradation of vesicle-associated α-synuclein, whereas ubiquitylation in ^96^K [36] and protein partners known to bind this region may prevent degradation by CMA of free soluble α-synuclein. Interestingly, the KFERQ-like motif is also masked in the structure of α-synuclein fibers (**Fig 2F**), supporting the previous experimental findings that, once in this oligomeric state, the protein is no longer amenable for CMA [8]. Checkpoint kinase 1 (CHK1) KFERQ-motifs and structure are depicted in **Fig 2G** as an example of how motifs in different protein domains could contribute to the degradation of functionally different forms of the same protein. Although structural information is only available for the kinase region of CHK1, it is well accepted that activation requires release of self-inhibition [37] through conformational changes dependent on phosphorylation of ^345^S, separated only by four amino acids from the canonical KFERQ-like motif in CHK1 (**Fig 2G**). Changes in the already-reported phosphorylation of the serine residues flanking the motif [38] may prevent or promote HSC70 recognition and subsequent degradation. In fact, we have previously reported that CHK1 degradation by CMA is modulated by phosphorylation [11]. Similarly, ubiquitylation/deubiquitylation events in the putative motif of the catalytic region may modulate degradation of the still inactive CHK1 (**Fig 2G**). Phosphorylation has also shown to be a triggering event in degradation of the lipid droplet-associated protein perilipin 3 (PLIN3) by CMA [12], which is a limiting step to initiate lipolysis. One of the PLIN3 motifs is in the perilipin/ADRP/TIP47 (PAT) region—used for association to lipid droplets—making it likely buried in the lipid surface (**Fig 2H**). Phosphorylation in two residues downstream of the motif (^106^S) may facilitate its exposure. In fact, phosphorylation of ^245^S right before the second motif—located in the beginning of the four-helix bundle region—has been described to induce a conformational change for interaction with the hormone-sensitive lipase that will initiate lipolysis [39]. It is attractive to propose that those conformational changes will modulate HSC70 access to that region and contribute to modulating PLIN3 degradation by CMA. The information on position and PTMs of KFERQ-like motifs provided in this work may help guide future experimental studies on regulated protein degradation.

### Analysis of amino acids within KFERQ-like motifs

In our effort to analyze all conceivable KFERQ-like motifs, we studied every amino acid permutation allowed within the rules that determine the architecture of the motifs (**Fig 1A**). However, some arrangements of amino acids may be better suited for binding to HSC70 than others. In light of the current lack of experimental information on the physical and structural basis for HSC70 binding, we decided to analyze if specific amino acid arrangements inside the KFERQ-like motif are found in the human proteome more frequently.

**Fig 3:**
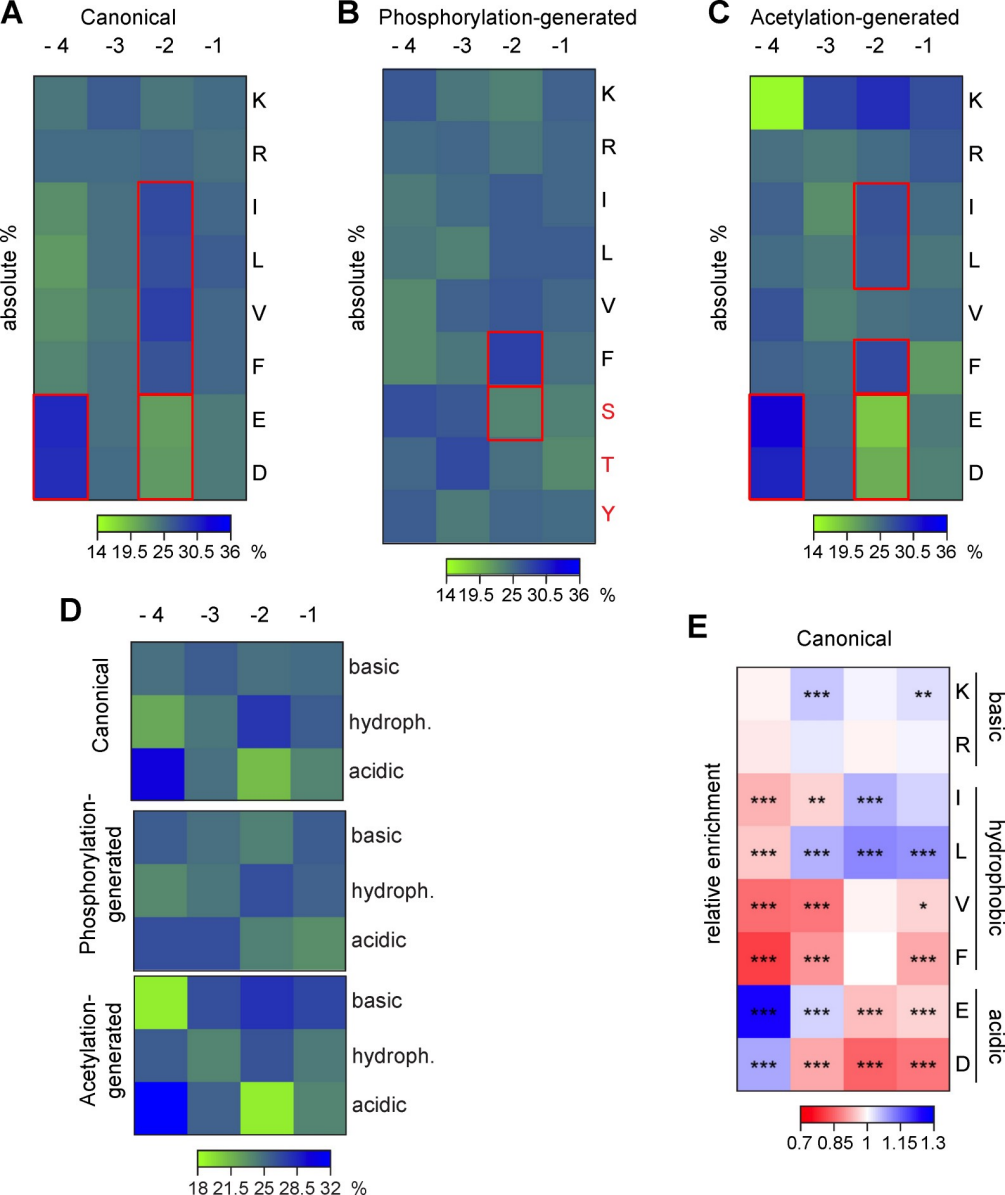
Amino acid positioning and frequencies within KFERQ-like motifs. (**A-C**) Frequency of amino acids at the four variable positions in canonical (**A**), phosphorylation-generated (**B**), and acetylation-generated (**C**) motifs in the human proteome. To allow superimposition, all motifs were aligned with a downstream glutamine. The amino acid positions are given relative to the glutamine (−1 = closest and −4 = furthest away). For each amino acid, the counts at each position are normalized as the percentage of the sum of all four positions. The phosphorylation acceptors serine, threonine, and tyrosine (red) are classified as acidic because they appear as an acidic residue once phosphorylated. Red boxes highlight consistent changes in abundance across motif types (see text for details). (**D**) Frequency of amino acids grouped by biochemical properties (basic, hydrophobic, acidic) at the four variable positions. The groups are the same three type of KFERQ-like motifs as shown in Fig 3A–3C. (**E**) Comparison of amino acid frequencies at each position in canonical motifs from the human proteome and from a permutated proteome. Amino acid counts from A are divided by the counts in motifs from permutated proteins. Means are from 40 random samples of 10% of the data sets each. ***p < 0.001, **p < 0.01, *p < 0.05. The *p*-values from two-sided *t* tests are corrected (Bonferroni) by the number of comparisons (*n* = 32). hydroph., hydrophobic;

We calculated for each amino acid its relative percentage at the four positions in the motif (because Q is in a fixed position). To allow superimposition, all motifs were aligned with a downstream glutamine independently of their original orientation in the protein. **Fig 3A** shows the results for the analysis of all canonical motifs in the human proteome. The relative percentage of hydrophobic amino acids was lowest at the position furthest away from the glutamine (<23%, position −4) and highest at position −2 (>27%). In contrast, the acidic amino acids were more frequent at position −4 (30%) and less commonly found in position −2 (21%). The basic amino acids showed no clear location preference within the motif. A similar analysis in putative motifs revealed that, in phosphorylation- (**Fig 3B**) and acetylation-generated motifs (**Fig 3C**), position −2 was still the most common for hydrophobic amino acids. Acidic residues in acetylation-generated motifs maintained similar preference for position −4 and low frequency in position −2, as observed in the canonical motifs (**Fig 3C**), while among the phosphorylation amenable amino acids used in phosphorylation-generated motifs, only serine showed a weak trend in this direction (**Fig 3B**). Despite some differences in relative frequency at each position, when grouped by categories, the higher frequencies of an acidic residue in −4 and a hydrophobic residue in −2 were still observed for all three types of motifs (**Fig 3D**). Overall, these findings suggest a more important role of both acidic and hydrophobic residues in motif construction. Their preferred placement in the motif’s borders is in agreement with our previous result from the prediction of solvent exposure that the more exposed amino acids are at the motif’s borders and the less exposed are in the motif’s core (**Fig 2C and 2D**).

To analyze if some amino acids preferentially occur in KFERQ-like motifs, we corrected their frequencies in the motifs by their relative abundance in the total proteome. Therefore, we generated a baseline for the amino acid frequency in KFERQ-like pentapeptides using motifs extracted from randomized sequences with the same amino acid composition as the human proteome. This comparison showed that leucine is enriched among hydrophobic residues both in the preferred and less common positions (15% increase, position −2), while the presence of valine and phenylalanine is below baseline (20% depletion, position −4) (**Fig 3E**). Among the acidic residues, glutamic acid is the most frequent amino acid at any given position (30% increase, position −4), and for the basic residues, lysine is slightly more enriched compared with arginine (**Fig 3E**). Similar trends are observed for phosphorylation- and acetylation-generated motifs, although the amino acid preferences for phosphorylation-generated motifs are less pronounced compared with the other kinds of motifs (**S3A and S3B Fig**). Importantly, the frequency of amino acids involved in the formation of KFERQ-like motifs is the same for proteins harboring a KFERQ-like motif as for proteins without a motif (**S3C Fig**). Future experimental studies are needed to test whether preference of amino acid usage and location in a KFERQ-like motif are predictive of HSC70 binding affinity.

### Evolutionary conservation of KFERQ-like motifs

**Fig 4:**
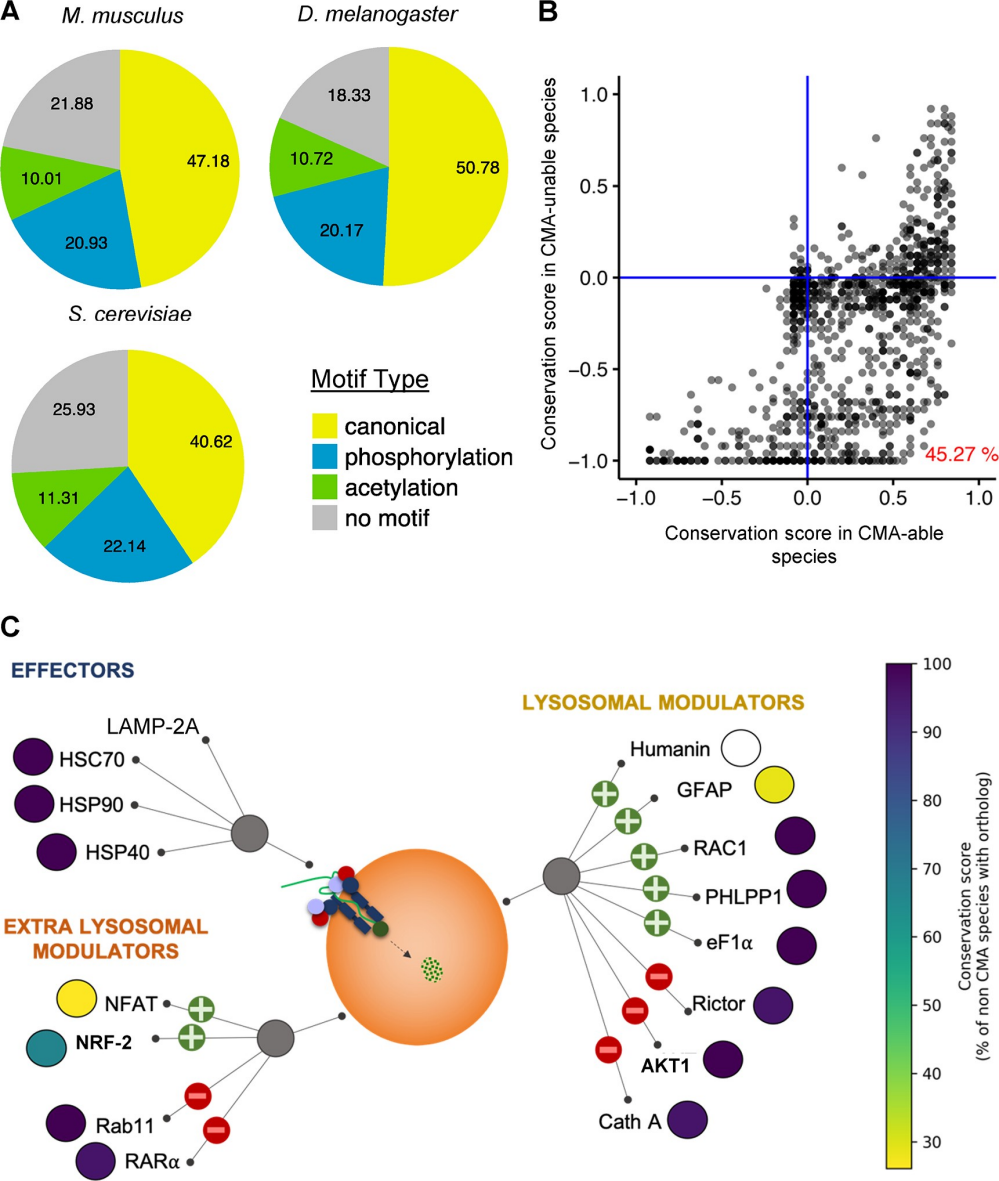
Conservation of KFERQ-like motifs and CMA components among species. (**A**) Percentage of proteins with the indicated types of KFERQ-like motifs in the referenced proteomes of *M*. *musculus*, *D*. *melanogaster* and *S*. *cerevisiae*. Only reviewed Swiss-Prot entries are included. The occurrence of motifs is ranked as canonical > phosphorylation-generated > acetylation-generated, and proteins with a combination of motifs are assigned to a group based on their highest-ranking motif. (**B**) Scatterplot of the conservation of motifs from human proteins with a single canonical motif in orthologs from the list of species predicted to be able or unable to perform CMA based on detection of LAMP-2A (S4C Fig). Sequences are aligned in MUSCLE (drive5.com/muscle) and motifs identified in the pentapeptides match the exact position of the human motif. The conservation score was calculated as follows: $\frac{n_{conserved}\;+\;0.5\;n_{partial}\;-\;n_{noOrth}}{n_{species}}$, where n_partial_ = species with motifs of a different type and *n*_*noOrth*_ = species with no ortholog identified. A conservation score >0 indicates that it is more likely than not to find an ortholog with a motif at the same position as the human protein. (**C**) Conservation of CMA machinery across species. Proteins involved in CMA are grouped based on their function (effector and modulators) and localization (lysosomal and extra-lysosomal). The colored disk next to the name of each element represents the conservation between CMA-able and CMA-unable species, as indicated by the lateral color bar. Positive and negative symbols indicate their function as activators or inhibitors of CMA activity. AKT1, RAC-alpha serine/threonine-protein kinase; Cath A, lysosomal protective protein/cathepsin A; CMA, chaperone-mediated autophagy; eF1α, Elongation factor 1-alpha; GFAP, Glial fibrillary acidic protein; HSC70, Heat shock cognate 71 kDa protein; HSP40, DnaJ homolog subfamily B member 1; HSP90, Heat shock protein HSP 90; LAMP-2A, lysosome-associated membrane protein type 2A; NFAT, nuclear factor of activated T cells; NRF-2, nuclear factor erythroid 2-related factor 2; PHLPP1, PH domain leucine-rich repeat-containing protein phosphatase 1; Rab11, Ras-related protein Rab-11; RAC1, Ras-related C3 botulinum toxin substrate 1; RARα, Retinoic acid receptor alpha.

In contrast with other types of autophagy conserved from yeast to mammals, CMA is of a relatively late evolutionary development [40]. The recent discovery that a different type of autophagy, eMI, also requires KFERQ-like motifs for protein degradation and the fact that, in addition to mammals, eMI has also been described as early as *Drosophila melanogaster* [41] make it impossible to establish a link between CMA and abundance of KFERQ-motifs in proteomes of different species. However, because LAMP-2A, the essential component of CMA, is absent in flies, rendering them unable of performing CMA, we still considered of interest comparing KFERQ-motifs in the proteomes of species with different CMA capability. Thus, we analyzed the proteomes of *Mus musculus* (capable of both CMA and eMI), *Drosophila melanogaster* (capable of eMI but not CMA), and *Saccharomyces cerevisiae* (unable to perform either). We found that, although these organisms differ in their ability to perform each of these types of selective autophagy, the overall percentages of proteins bearing each of the types of KFERQ-like motifs are comparable (**Fig 4A**). We noticed, however, a trend towards a higher proportion of proteins with KFERQ-like motifs, specifically canonical ones, in species that could perform at least one of these types of autophagy (**Fig 4A**). Studies targeted to identify if HSC70 binds to proteins with motifs in *S*. *cerevisiae* and the fate of those proteins could help in identifying an alternative to these selective forms of autophagy in yeast.

To begin to systematically gather similar information across additional species, we grouped them based on their potential to perform CMA. Because CMA depends on the presence of a spliced variant of the *LAMP2* gene, we took advantage of the two unique features in the C-terminus of this variant that differentiates it from the other two LAMP2 isoforms in mammals: (1) the presence of three to four basic amino acids in the proximal region of the C-terminus cytosolic tail [42] and (2) the sequence GYEQF at the end of the C-terminus (**S4A Fig**). Furthermore, because experimental studies have demonstrated that the addition of a short stretch of amino acids (i.e., a 7–amino acid hemagglutinin tag) to the C-terminus of LAMP-2A was enough to disrupt its CMA ability [42], we also imposed the criterion that GYEQF-homology sequences should be present at the end of the C-terminus. Using the Basic Local Alignment Search Tool (BLAST) we searched for homologous sequences to the LAMP-2A tail that conform to these criteria and found that the presence of LAMP-2A isoforms is limited to mammalian species together with some bird [40] and reptile species (**S4B Fig**). Note that although a recent theoretical commentary proposes that CMA occurs in some types of fish, based on the sequence analysis of a spliced variant of the *LAMP2* gene containing GYEQF followed by an additional amino acid [43] (**S4C Fig**), we decided against allowing that variation in our search criteria until experimental evidence that this isoform is required for CMA is generated. Intriguingly, not all branches within the class Mammalia possess LAMP-2A. For example, no homologous sequences were found in Metatheria indicating that these may constitute a separate line in the development of selective autophagy.

Using this information in combination with a list of organisms for which rich proteomic information is available (treefam.org, March 2013) [44], we constructed a set of 50 species classified by the presence or absence of a LAMP-2A homologue that we generically named CMA-able or CMA-unable, respectively (**S4C Fig**). Orthologs of human proteins harboring a single canonical KFERQ-like motif were identified using the EggNOG database of orthologous groups (eggnogdb.embl.de), and all sequences of those orthologs within the 50 CMA-able or -unable species were selected. After alignment of the sequences, the positions corresponding to the KFERQ-like motif in the human sequence were analyzed for the presence of a CMA-targeting motif. A conservation score was calculated for the species grouped by their capability to perform CMA to determine which motifs are selectively conserved in species with CMA. We found that about 45% of the investigated motifs are more conserved in CMA-proficient species over CMA-deficient species (**Fig 4B;** a conservation score above zero indicates that it is more likely to find an ortholog with a motif at the same position as the human protein than not). In CMA-unable species, the score is often negative because many of them have no clear orthologs to human proteins. However, no motif is fully conserved in CMA-able species either. When setting a score of >0 (more than average conservation) as threshold, the motifs of 503 proteins are selectively conserved in CMA-able species and not in CMA-unable ones (**S3 Table**). We speculate that this difference in conservation suggests a higher number of true positive KFERQ-like motifs in this group. Interestingly, analysis of the frequency of amino acids involved in the formation of KFERQ, as in **S3C Fig** but across species, revealed that amino acid percentages are also conserved among CMA-able species, while the percentages are noticeably more variable for the less closely related group of species unable to perform CMA (**S3D Fig**).

To estimate the conservation of other CMA components (effectors and regulators) across evolution, we used the division on CMA-able and CMA-unable species established from the conservation analysis of the LAMP-2A cytosolic tail. We found that 73% of CMA components (including core machinery [effectors] and lysosomal and extra-lysosomal regulators) are highly conserved across all species (**Fig 4C** and **S4 Table**). Only two of the extra-lysosomal CMA regulators (nuclear factor of activated T cells [NFAT] and nuclear factor erythroid 2-related factor 2 [NRF-2]) and two of the lysosomal regulators (Glial fibrillary acidic protein [GFAP] and Humanin) showed partial conservation (**Figs 4C** and **S4C** and **S4 Table**). This is in line with the hypothesis that the LAMP-2A isoform plays a critical role for CMA and its absence is sufficient to make a species unable to perform CMA.

### Enrichment of KFERQ-like motif classes in functional terms

When considering the coexistence of both constitutive and posttranslational-generated KFERQ-like motifs in a given proteome, a possible prediction would be that proteins with buried motifs (either in their structure or through protein–protein or protein–membrane interactions) will be more likely to have canonical motifs, whereas proteins in which the motif is in an easily accessible region may use generation of motifs through PTMs to prevent continuous degradation by CMA. As a consequence, we expect that proteins harboring particular motifs would be better suited for some but not other cellular processes.

To analyze if different types of KFERQ-like motifs associate with specific cellular processes, we performed an enrichment analysis using biological process annotations from gene ontology (GO; geneontology.org, **S5 Table**). Applying the previously mentioned motif hierarchy (first, canonical; next, phosphorylation-generated; followed by acetylation-generated motifs; **Fig 1B**), we observed clear differences in the biological processes enriched in each type of motif (**S5A Fig** and **S6 Table**). Canonical motifs are highly enriched in cytoskeleton-associated terms (cytoskeleton organization, cell projection organization), proteins with phosphorylation-generated motifs are frequently associated with transport-related terms, and proteins with acetylation-generated motifs with metabolism-related terms. Strikingly, the group of proteins without a CMA-targeting motif shows only weak enrichment in biological processes, and we confirmed that this is not an effect of particularly low annotation density in this group (**S5B Fig**). To further expand the analysis of a possible association between KFERQ-like motifs and cellular functions, we performed a similar analysis of hierarchy-arranged motifs with a protein localization database (COMPARTMENTS, https://compartments.jensenlab.org) and compared the cellular distribution of KFERQ-bearing proteins with the full proteome distribution. Interestingly, we found significant enrichment of proteins bearing canonical motifs in cytoskeleton, cytosol, and endosomes and of proteins with phosphorylation-generated motifs in mitochondria, when compared with the overall proteome distribution (**S5C Fig and S6 Table**). Also, we noted a significant decrease of KFERQ-like motifs of any type in proteins in the extracellular space (**S5C Fig and S6 Table**).

**Fig 5:**
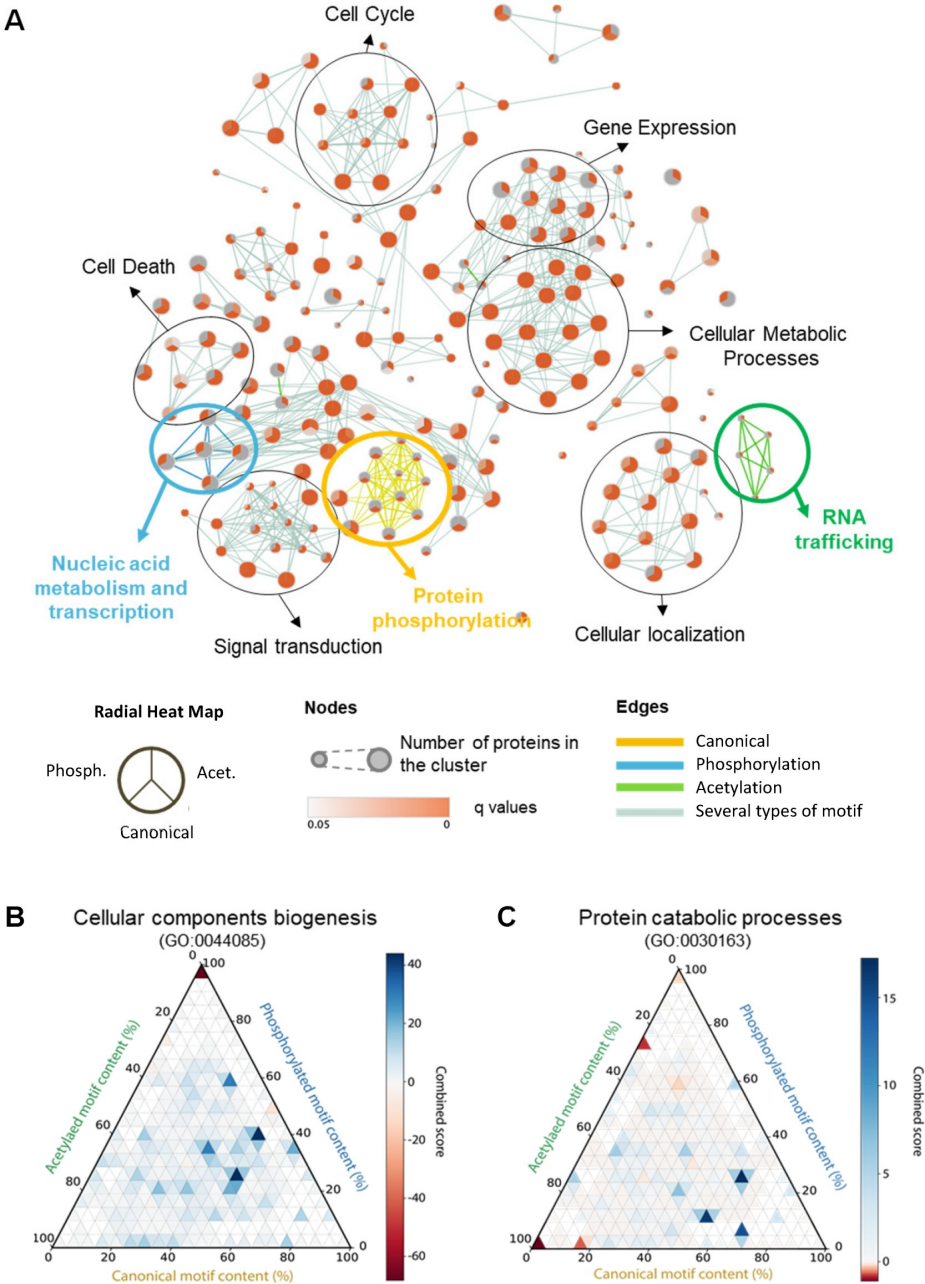
Enrichment of proteins with KFERQ-like motifs in biological processes. **(A)** Enrichment map analysis of the association of proteins with KFERQ-like motifs with biological processes. The nodes are radial heat maps in which size shows the number of proteins within the given annotation and intensity of color indicates association with a specific kind of motif (the position of each type of motif in the radial heat map is indicated in the legend). Edges represent similarity between nodes, and color coded circles indicate clusters associated with a specific motif type (yellow for canonical, blue for phosphorylation-generated, and green for acetylation-generated motifs). (**B,C**) Enrichment for human proteins annotated under cellular components biogenesis (**B**) and under protein catabolic processes (**C**), grouped by relative content of KFERQ-like motif classes. For each protein, the fractional content (0% to 100%) of canonical, phosphorylation-, and acetylation-generated motifs is calculated. Proteins are binned by motif composition using a 5% bin size for each dimension. The combined score for enrichment (−log_e_(*p*-value)*z-score) of the proteins in each bin (small triangles within the plot area) is color coded from blue (low) to red (high). Acet., acetylation; Phosph., phosphorylation.

The unexpected high percentage of the proteome that contained more than one KFERQ-like motif (**Fig 1C**) made us consider whether specific combinations of motifs may also be associated with some biological process. Further analysis of the enrichment of the biological process in proteins grouped according to their combinations of motifs confirmed that this was indeed the case and that some combinations of motifs are more frequently found together in certain functional terms. For example, inside the group of proteins with canonical motifs, those also bearing an acetylation-generated motif were enriched in cytoskeleton-associated processes (**S5D Fig** and **S7 Table**). This observation motivated us to perform a network analysis of GO term enrichment, but this time without applying a hierarchy for motif types. Using this strategy, we could show that several cellular functions (i.e., cell cycle, gene expression, signal transduction, cellular localization, cellular metabolic processes, and cell death) formed clusters associated with all kind of motifs (**Fig 5A**, black circles). Interesting, a small subset of clusters was associated with unique motif types (**Fig 5A,** color coded circles; and **S6A–S6C Fig** for higher magnification). Thus, proteins with canonical motifs were associated with protein phosphorylation (including both regulation of kinase enzymes activity and of phosphate metabolism) (**S6A Fig**) and proteins with phosphorylation-generated motifs associate with nucleic acid metabolism and transcription (**S6B Fig**), whereas proteins with acetylation-generated motifs associated with RNA transport and localization (**S6C Fig**). These findings strengthen the idea that functionally related proteins may also share common signals for their regulated selective degradation by CMA.

In the case of proteins with multiple motifs, we also calculated the exact motif composition expressed as a percentage of each motif type for different biological processes. For example, a protein with two canonical, one phosphorylation-, and one acetylation-generated motifs will be assigned a fractional content of 50% canonical, 25% phosphorylation-generated, and 25% acetylation-generated motifs. To display the enrichment of proteins grouped by their fractional content, we utilized triangle plots that display the three motif dimensions in a two-dimensional space (as illustrated by the cartoon in **S7A Fig**). This method confirmed the association of some GO terms with particular multiple-motif combinations. For example, in the case of proteins with at least one canonical motif, the term “cellular component biogenesis” was enriched in proteins with a generally high content of canonical motifs and intermediate content of phosphorylation-generated motifs (**Fig 5B**). In comparison, proteins participating in protein catabolic process had a higher ratio of canonical and acetylation-generated and fewer phosphorylation-generated motifs (**Fig 5C**). In the case of proteins with only putative motifs, terms like transmembrane transport were clearly associated with phosphorylation-generated motifs (**S7B Fig**), whereas proteins involved in mRNA catabolic process were enriched in acetylation-generated motifs (**S7C Fig**). These results support an association between the type and combination of KFERQ-like motifs in proteins and their participation in specific biological processes, thus suggesting a differential regulatory role for CMA in biological function through this elaborated code of protein targeting motifs.

### The KFERQ-finder computational tool

To allow other researchers to utilize these analyses for their work, we have developed the “KFERQ finder” (**S8 Fig**), a free web-based tool for performing KFERQ-like motif searches in proteins of interest. Protein sequences are input using their UniProt ID or as FASTA sequence, and the tool searches them against all possible pentapeptides containing a KFERQ-like motif. The “KFERQ finder” menu gives the possibility of (1) analyzing individual or in-bulk inputs, (2) selecting the type of KFERQ-like motif to search for, and (3) allowing additional options such as advanced motifs (to include replacement of Q by N) or inactivation of the motif through ubiquitylation. We recommend caution when using the Q by N replacement search because, as indicated in the previous sections, those motifs may need still unknown features to lead to CMA targeting (they are often necessary but not sufficient). The results are presented as (i) entry name (UniProt ID/sequence name), (ii) status of the protein in the UniProt Database, (iii) protein names, (iv) gene name, (v) protein length, (vi) motif composition, (vii) motif position (start amino acid), and (viii) type of motif (“KFERQ finder,” **S8 Fig**).

To validate the use of this tool for the identification of KFERQ-containing proteins and potential CMA substrates, we experimentally analyzed the degradation properties of proteins identified in silico by the search tool as having KFERQ-like motifs but never previously reported as CMA substrates. As input in the KFERQ finder, we used a list of proteins for which we had access to antibodies (>200) and randomly selected six proteins bearing a KFERQ-like motif and three without motif to analyze their degradation (**S9 Fig**). We treated NIH3T3 cells with inhibitors of lysosomal proteolysis (NH_4_Cl and leupeptin) to determine the fraction of protein undergoing lysosomal degradation and used NIH3T3 cells knocked down (KD) for LAMP-2A to analyze the CMA dependence of their degradation (**S9A Fig**). Glyceraldehyde-3-phosphate dehydrogenase (GAPDH), a well-characterized CMA substrate, was included as positive control, and the efficiency of lysosomal proteolysis inhibition was confirmed by measuring the degradation of microtubule-associated proteins 1A/1B light chain 3B (LC3-II) that takes place via macroautophagy (**S9B Fig**). We confirmed that GAPDH and the other five KFERQ-bearing proteins all underwent lysosomal degradation, albeit at different rates (**S9A Fig**). Degradation of three of the new proteins was dependent on LAMP-2A (1) (lysosomal degradation through CMA), whereas two of the new KFERQ-containing proteins still underwent degradation in LAMP-2A KD cells (2) (their lysosomal degradation could instead be mediated through eMI, which also utilizes this targeting motif) (**S9A Fig**). None of the proteins that rendered negative for the presence of KFERQ-like motif in their sequence were degraded in lysosomes (3) (**S9A Fig**). Although additional experiments are needed to fully confirm that the new proteins are bona fide CMA substrates (i.e., by eliminating the KFERQ-like motif or reproducing their direct transport into isolated lysosomes [45]), this experiment validates the use of the KFERQ finder to identify putative CMA substrates.

## Discussion

The presence of a KFERQ-like motif in a protein is necessary and sufficient for its targeting for degradation via CMA [2]. In this study, we performed the first proteome-wide search for KFERQ-like motifs in the human and additional proteomes. We have made the tool developed in this work for identification of canonical and putative KFERQ-like motifs in proteins publicly available on the website “KFERQ finder.” This tool allows for motif searching in individual proteins, protein groups, and sequences directly pasted in the site and depicts the types of identified motifs and their location in the protein sequence (**S8 Fig**). The KFERQ finder integrates all the necessary steps to identify KFERQ-like motifs, provides a reliable source for researchers without requiring in-depth knowledge about the rules generating motifs, and should help to further prevent populating the scientific literature with erroneous motifs, as has already happened.

Our analysis of the abundance, positioning, local environment, and amino acid composition and conservation of the KFERQ-like motif has revealed that about 75% of proteins in the human proteome are in principle amenable for degradation via CMA, as they contain at least one canonical or one putative KFERQ-like motif. The breakdown according to type of motif demonstrates occurrence of canonical KFERQ-like motifs in about 45%–47% of proteins in the human proteome. This percentage is slightly higher than the 30% of soluble cytosolic proteins previously pulled down with an antibody generated against the original KFERQ pentapeptide [46]. However, the fact that only cytosolic proteins were used for the pull down assays, whereas recent studies have shown that proteins resident in other compartments (i.e., nucleus, mitochondria, lipid droplets) can undergo CMA degradation once they reach the cytosol [11] makes us consider our current estimation of total canonical motifs in the proteome more accurate.

The percentage of proteins with KFERQ-like motifs is relatively similar across species, even in those species that do not have CMA, like yeast. It is thus possible that the KFERQ is an ancient motif for HSC70 binding to proteins for other functions and that later in evolution, it was repurposed as a targeting motif for CMA. In fact, except for LAMP-2A and some of the CMA regulators, most of the proteins described as CMA components (effectors and regulators) are conserved across species independently of their ability to perform CMA. This finding further highlights the lysosomal receptor LAMP-2A as an essential CMA component, and the possible evolutionary relation between CMA and other autophagic pathways, such as eMI, that share the targeting motif and the chaperone with CMA [47]. The main difference when comparing KFERQ-like motifs between a species with no CMA or eMI (such as yeast) with one with eMI but lacking CMA (such as flies) was an increase in the percentage of proteins bearing a motif (from 74% to 81%), which was mostly due to an increase in canonical motifs. The introduction of CMA as an additional pathway in mammalian species did not further increase the percentage of proteins bearing a motif (in fact, there was a slight 3%–6% increase in proteins without motif), and the abundance of each of the types of motifs remained unchanged. These results suggest that rather than generating new variations of motifs for CMA targeting, the same motifs used for eMI in flies were used for both eMI and CMA in species active for both pathways. Interestingly, although the overall percentage of proteins containing motifs was not so different between species capable or not of performing CMA, analysis of motif conservation in orthologous groups of proteins demonstrated that more than 40% of motifs in proteins harboring a single canonical CMA-targeting motif are highly conserved in CMA-able species but not conserved in CMA-unable species. The high conservation of these motifs suggests that they are likely preferred for targeting proteins towards CMA. Further studies on the evolution of eMI should help to confirm or refute this statement.

Previous studies experimentally tagging KFERQ-like motif sequences to non-CMA substrate proteins demonstrated similar CMA efficiency for N- or C-terminus–positioned tags [5, 48]. In agreement with these findings, we found no strong preference for the position of KFERQ-like motifs along the protein length apart from a decreased number of motifs close to the N-terminus. However, we found position preference for certain amino acids within the motif, with acidic residues more often located in the farthest position from the glutamine and hydrophobic amino acids more frequently observed as the second residue from the terminal glutamine. The current low number of experimentally confirmed CMA substrate proteins makes it difficult to accurately determine similar position preferences. Results from ongoing studies on the interacting surfaces between HSC70 and the KFERQ-like motif in different proteins and the anticipated increase in experimentally confirmed motifs in the future should help to further refine amino acid position preference.

In both experimentally validated CMA substrates and in proteins identified to harbor a single canonical motif, the amino acids at the edges of the motif have a striking increase in the probability of being exposed to the surrounding medium. In contrast, the amino acid at the motif center has a tendency to be more buried, and this observation is mirrored by our proteome-wide prediction of the preference for acidic amino acids to be located at the opposite side of the glutamine and hydrophobic amino acids to be more often found in the middle of the motif. We propose that the flanking amino acids of the motif may be the sites of initial contact with HSC70 that then could use its unfoldase activity to further expose the other motif residues.

Accessibility of the KFERQ-like motif to HSC70 can also depend on the properties of the protein domain where the motif resides and on its amenability to undergo PTMs. For example, we found a high frequency of KFERQ-like motifs in domains used for protein–protein interaction. Similarly, we provide examples of previously described protein PTMs that could favor or prevent HSC70 binding to the motif.

The proteome-wide identification of KFERQ-like motifs performed in this work also allowed us to start investigating the possible linkage of types of motifs with specific cellular functions. Beyond the clear enrichment of proteins in cellular pathways depending on whether they had a canonical or putative motif in their sequence, we found that specific combinations of motifs coexisting in the same protein were also enriched in particular biological processes and specific cellular locations. Future studies are needed to determine whether each of the multiple motifs present in the same protein are utilized to modulate their CMA degradation under different conditions or if they are used for degradation of different conformations of the same protein.

As indicated in previous sections, in some instances, an asparagine (N) can substitute for the flanking glutamine (Q) in the motif. Recognition of the N-bearing motifs by HSC70 has been demonstrated experimentally in proteins such as GAPDH or hypoxia-inducible factor 1-alpha (HIF1α) [9, 24, 49]. However, contrary to the Q-bearing motifs that are necessary and sufficient for HSC70 binding, motifs with N instead of Q are necessary but not sufficient for HSC70 binding when attached to nonsubstrate proteins (F. Dice, personal communication). This class of motifs was not included in the systematic approach of this study because of the current lack of information on the additional determinants that contribute to HSC70 recognition. However, in order to facilitate future studies to identify these common determinants, we have included the possibility of searching for N-bearing motifs in the KFERQ-finder software. Using that tool, we have performed a search in the human proteome for proteins bearing only one of these motifs to provide initial insights on the abundance, preferred location, and general functions of proteins containing N-bearing motifs only (**S10 Fig and S8** and **S9 Tables**).

The new search tool developed in this study could guide hypothesis generation for the possible involvement of CMA in specific cellular functions. For example, the abundance of proteins containing KFERQ-like motifs in DNA repair pathways triggered the studies that identified a function for CMA in genome maintenance [11]. Furthermore, comparison of changes in levels of KFERQ-containing proteins in proteomes of experimental disease models or patients could inform on the contribution of CMA malfunction to the pathogenesis of the disease.

We anticipate that use of the freely available “KFERQ finder” software, developed in this work, by the scientific community will not only accelerate discovery and validation of new CMA substrates, thus expanding understanding on the physiological relevance of this type of autophagy, but will also assist in guiding further refinement of the motif itself.

## Materials and methods

### Software and data resources

The *Homo sapiens* (UP000005640), *M*. *musculus* (UP000000589), *D*. *melanogaster* (UP000000803), and *S*. *cerevisiae* (UP000002311) proteomes were downloaded from UniProt.org (retrieved January 16, 2018). Data analyses were performed using R [34] (3.3.2) together with the packages dplyr [50] (0.7.4), stringr [51] (1.2.0), gplots [52] (3.0.1), ggplot2 [53] (2.2.1), jsonlite [54] (1.5), and bio3d [55] (2.3–3). Protein structures were obtained from the RCSBPDB protein data bank (rcsb.org) and PTMs from the PhoshoSitePlus [56]. All code used for analyses can be found at https://github.com/PhilippKirchner/KFERQ_analysis.

### Analysis of amino acid frequencies in KFERQ-like motifs

The frequency for each amino acid at the four variable positions in the motifs (shown as percentage of total counts) was calculated and compared with a baseline frequency calculated for motifs identified in the same proteome after scrambling each protein sequence. Means and standard deviations were derived by repeatedly (40 times) sampling 10% of each data set. Statistical tests were performed using two-sided *t* tests with a Bonferroni correction of the *p*-values for multiple testing (*n* = 32).

### Prediction of solvent exposure from primary sequence

The relative solvent exposure was calculated from crystal structures (pdb.org) or predicted from amino acid sequence using JPred4 [31] (compbio.dundee.ac.uk/jpred). JPred4 accepts inputs with a maximum length of 800 amino acids. Therefore, all proteins with a length above this limit were removed from the analysis to avoid unknown behavior of truncated input sequences. An amino acid with a relative solvent accessibility <0.25 was considered buried.

### Identification of LAMP-2A isoforms

Isoforms of the LAMP-2A protein were identified in a Protein BLAST (https://blast.ncbi.nlm.nih.gov/Blast.cgi) search using the last 100 amino acids of each protein in the human proteome. The results were then filtered using the regular expression “[K,R,H]{3,4}*{1,3}GYEQF$” to match the required features of the LAMP-2A C terminus tail.

### Alignment of orthologs and calculation of conservation scores

Using the information of LAMP-2A expression, species from different branches of the phylogenetic tree (treefam.org) were classified as CMA-able and CMA-unable. For the analysis of motif conservation across species, orthologs were identified from the EggNOG database (eggnogdb.embl.de) for a set of human proteins with one canonical motif. Species from the treefam.org database not well represented in the EggNOG database were manually corrected to a better-covered subspecies of the same species when possible. If more than one possible ortholog was returned, the two with the highest alignment score (minimum expectation value and maximum alignment score, BLAST) were selected. All orthologs for a specific protein were aligned using MUSCLE [57] (https://www.drive5.com/muscle/), and motifs were identified in the pentapeptide matching the motif position in the human protein. After identification of motifs in the isolated pentapeptides, the conservation score was calculated as follows: $\frac{n_{conserved}+0.5\;n_{partial}-\;n_{noOrth}}{n_{species}}$, where n_partial_ = species with motifs of a different type and *n*_*noOrth*_ = species with no ortholog identified.

### Analysis of association with biological processes

GO terms were manually selected using the PANTHER GO-slim set (http://www.pantherdb.org/panther/ontologies.jsp) as a starting point to cover a wide range of intracellular processes with general terms. To improve annotation density, annotations for all available GO terms were mapped to the custom group using the map2slim algorithm (go.princeton.edu/cgi-bin/GOTermMapper). For each group of proteins, enrichment probabilities were calculated using the Fisher exact test. Z-scores were calculated by repeated (40 times) random resampling of GO term annotations. The combined score was then derived as −log_e_(*p*-value)*z-score. Ternary plots [58] were made using the python-ternary package in Python 3.7 and the Python scientific stack [59, 60], colbert [61]. Enrichment map analysis was performed using Cytoscape 3.7.1 [62] and Enrichment map 3.1.0 [63].

### Analysis of protein degradation

NIH-3T3 mouse fibroblasts (American Type Culture Collection, Manassas, VA, ATCC CRL-1658) were cultured in a 37°C incubator with 5% CO_2_ in DMEM supplemented with 10% newborn calf serum. For lysosomal proteolysis assays, a combination of two lysosomal proteolysis inhibitors (lys inh), ammonium chloride (20 mM; Sigma-Aldrich, St. Louis, MO, A9434) and leupeptin (100 μM; Fisher Scientific, Hampton, NH, BP26621), were added directly to the media, and cells were incubated for 12 h or 24 h (as indicated) in serum-free media to induce CMA activity. At the end of the incubation, cells were lysed in 0.25 M sucrose buffer (pH 7.2) supplemented with protease inhibitors, and samples were subjected to electrophoresis and immunoblot in nitrocellulose membranes. Sources of primary antibodies and dilutions used were as follows: against LC3 (1:1,000; Cell Signaling, Danvers, MA, 3868), mouse LAMP-2A (1:3,000, Thermo Scientific, Whaltman, MA, 512200), Alix (1:1,000, Cell Signaling, 2171S), CYLD (1:1,000; Sigma-Aldrich, SAB4200060), ATF6a (1:1,000, Novus Biologicals, Centennial, CO, nbp1-40256), ACACA (1:1,000, Cell Signaling 3676), ATGL (1:1,000, Cell Signaling, 2138), CDKN2A (1:1,000, Abcam, Cambridge, UK, ab109199), H2A.X (1:1,000, Cell Signaling, 2595), BCL2 (1:1,000, Cell Signaling, 2870), PSA5 (1:1,000, Biomol GmbH, Hamburg, Germany, pw8125), and GAPDH (1:1,000, Abcam, ab8245). The proteins of interest were visualized by chemiluminescence using peroxidase-conjugated secondary antibodies in G-BOX Chemi XX6 (Imgen, Alexandria, VA), and red ponceau was used as loading control. Sources of other chemicals were as described before [12, 18].

### Data availability

All raw data (individual numerical values that underlie the summary data displayed in main and supplementary figure panels) have been deposited in the publicly available repository, GitHub, and can be accessed through this link: https://github.com/PhilippKirchner/KFERQ_analysis/tree/master/raw_data_figures.

## Supporting information

S1 Fig Frequency of KFERQ-like motifs in additional human data sets. (**A**) Comparison between the length of proteins in the reviewed Swiss-Prot and complete UniProt human proteome. Swiss-Prot entries were filtered from the human proteome by their revision status in the UniProtKB. The density of proteins with a length <2,500 amino acids is shown. (**B**) Percentage of proteins with the different types of KFERQ-like motifs in the unfiltered human proteome. The data are grouped and displayed as in **Fig 1B**. (**C**) Distribution of the number of motifs per protein. For each protein, the number of motifs following the hierarchical priority described in **Fig 1B** was calculated. The bars in the histogram are colored according to the motif types.
(TIF)

S2 Fig Distribution of KFERQ-like motifs within protein sequences of experimentally confirmed CMA substrates. Absolute (**A**) and relative (**B**) positions of the KFERQ-like motifs in experimentally validated CMA substrates (taken from published literature summarized in [4]). The position and type of motif are indicated by colored boxes (yellow, canonical; blue, phosphorylation-generated; green, acetylation-generated). Red boxes indicate experimentally validated canonical motifs in which an N is found in place of a Q (these motifs are not included in the proteome-wide analysis because, in contrast to the other motifs, they require additional unknown circumstances to target a protein towards CMA). (**C**) Histogram of the frequency of canonical motifs along the protein length excluding initiator methionine residues. The data are presented as in Fig 2A. Red line indicates the slope of the reduction in KFERQ-like motifs. (**D**) Distribution of phosphorylation- or acetylation-generated motifs along the protein length. The length of the proteins is normalized to a scale from 0 (N-terminus) to 1 (C-terminus). The histogram shows the count of motifs at the relative position with a bin size of 0.02. (**E**) Examples of protein secondary structure analyses in validated CMA substrates. The relative solvent exposure of amino acids was calculated from pdb crystal structures or predicted using JPred4. Amino acids with a relative solvent exposure below 25% were considered buried (note that for RND3 and STING, pdb data were only available for a fragment of the protein, shown here aligned with the full sequence). The vertical yellow lines indicate the positions of the KFERQ-like motifs (the central amino acid of a motif marks the motif position). CMA, chaperone-mediated autophagy.
(TIF)

S3 Fig Amino acid frequencies within putative KFERQ-like motifs. Comparison of amino acid frequencies at each position in phosphorylation-generated (**A**) and acetylation-generated (**B**) motifs from the human proteome and from a permutated proteome. Amino acid counts from Fig 3B and 3C were divided by the counts in motifs from permutated proteins. To superimpose motifs starting or ending with a glutamine, motifs starting with a glutamine are mirrored. The amino acid positions are given, relative to the glutamine (−1 = closest and −4 = furthest away). Means are from 40 random samples of 10% of the data sets each. ***p < 0.001, **p < 0.01, *p < 0.5. The *p*-values from two-sided *t* tests are corrected (Bonferroni) by the number of comparisons (*n* = 32). (**C**) Frequency of total amino acids in proteins containing KFERQ-like motifs and proteins without a motif. For each protein in the unfiltered human data set, the percentage of amino acids that can become part of a KFERQ-like motif was calculated. The data set was then split into the pool of proteins with and without KFERQ-like motifs. The heat map displays the amino acid percentages in each group. (**D**) Amino acid frequencies calculated as in (**C**) but over the whole proteomes of species with (LAMP-2A+ = able to perform CMA) and without (LAMP-2A− = unable to perform CMA) the CMA receptor LAMP-2A. The analysis for presence of LAMP-2A in different species is presented in detail in **S4C Fig**. Amino acid percentages are scaled to standard normal distributions over the heat map columns to normalize differences in the relative abundance of individual amino acids. CMA, chaperone-mediated autophagy; LAMP-2A, lysosome-associated membrane protein type 2A.
(TIF)

S4 Fig Classification of species based on their predicted ability to perform CMA. (**A**) C-termini of LAMP-2A isoforms in species with experimentally demonstrated CMA activity and regular expression for identification of LAMP-2A homologues. (**B**) Species with a homologue to human LAMP-2A identified by a BLAST search against the C-terminal (100 amino acids) region and further filtered for exact matches to the pattern of the human LAMP-2A C-terminus. If multiple hits are returned, the one closest in length to human LAMP-2A is chosen. The evolutionary relation between all species with LAMP-2A homologues is shown as an evolutionary tree (visualization from itol.embl.com). Sub-trees for interesting nodes (Sauria, Aves, Mammalia, Rodentia, and Primates) are color coded. (**C**) Set of species used in the analysis of the evolutionary conservation of motifs. Organisms with available protein sequence information are selected from the TreeFam species tree (March 2013, treefam.org). In rare cases in which few EggNOG orthologs were found for a particular species from the treefam.org database, this conflict was manually resolved by selecting a different subspecies of this species with higher coverage in the EggNOG database, when available. The list of species is combined with the analysis of LAMP-2A to construct a set of 50 species classified by the presence or absence of a LAMP-2A homologue. Species with unclear status (e.g., containing imperfectly matching LAMP-2A tails) are omitted to simplify the classification. Upon analysis of the conservation of other components of the CMA machinery (**Fig 4C**), three proteins, GFAP, NFAT, and NRF2, showed only partial conservation but were selectively enriched in CMA-able species. The presence of these proteins in a species is indicated through color coded triangles. BLAST, Basic Local Alignment Search Tool; CMA, chaperone-mediated autophagy; GFAP, Glial fibrillary acidic protein; LAMP-2A, lysosome-associated membrane protein type 2A; NFAT, nuclear factor of activated T cells; NRF2, nuclear factor erythroid 2-related factor 2.
(TIF)

S5 Fig Enrichment of proteins with KFERQ-like motifs in biological process annotations. (**A**) Enrichment for a custom selected group of GO terms for biological processes (**S5 Table**) in human proteins, grouped by motif type (as in **Fig 1B**) or with no motif. For each group, the five most enriched terms (by combined score = −log_e_(*p*-value)*z-score) are displayed. Numbers on top indicate the total number of proteins in each group and numbers in the bars the percentage of motif-containing proteins in the proteins annotated for each term. See **S7 Table** for additional details. (**B**) Number of GO annotations per protein in the groups analyzed in **Fig 5A**. For each protein, the number of GO terms annotated in the UniProt database is calculated. The box plots show median, 25th, and 75th percentiles. Whiskers are 1.5 * IQR, and diamonds show the data set mean. Outliers are omitted for clarity. (**C**) Enrichment of KFERQ-like motifs in different compartments. A highlighted compartment indicates statistically significant difference from the whole proteome (chi-squared, *p < 0.05). Detailed statistics can be found in **S6 Table**. Red arrows show the most enlarged compartment. (**D**) Enrichment for a custom selected group of GO terms for biological processes (**S5 Table**) in human proteins grouped by combinations of canonical, phosphorylation-, or acetylation-generated motifs. For each group, the five most enriched terms are displayed. See **S7 Table** for additional details. GO, gene ontology.
(TIF)

S6 Fig Cluster analysis of KFERQ-like motifs with biological process annotations. **(A-C)** Clusters of GO terms associated with specific types of motifs: (**A**) canonical, (**B**) phosphorylation-generated, and (**C**) acetylation-generated. The nodes are radial heat maps in which the size is proportional to the number of proteins within the given annotation and the color intensity of the filling depicts association with a specific kind of motif (distribution indicated in the bottom of the figure). Edges represent similarity between nodes. GO, gene ontology.
(TIF)

S7 Fig Enrichment of proteins with mixed KFERQ-like motif content in biological process annotations. (**A**) Cartoon explaining the triangle plots used to display protein groups characterized by fractional motif content. For each protein, the fraction (0 to 100 percent) of canonical, phosphorylation-, and acetylation-generated motifs is calculated. Each side of the triangle plot represents the fraction of one motif class in 5% steps. The smaller triangles in the plot are bins of proteins with a specific motif combination. For example, proteins with a high content of canonical motifs will appear in the lower right corner (yellow), with proteins containing exclusively canonical motifs at the extreme right. The same is shown for phosphorylation-generated (blue) and acetylation-generated (green) motifs. Locations of hypothetical examples of motif composition percentages are shown with red lines. (**B, C**) Examples of GO terms showing enrichment for phosphorylation-generated (**B**) and acetylation-generated (**C**) motifs. GO, gene ontology.
(TIF)

S8 Fig The KFERQ-like motifs search tool. Screenshot of the free online software “KFERQ finder” for analysis of KFERQ-like motifs in protein sequences. Steps and options for database analysis are shown.
(TIF)

S9 Fig Experimental validation of KFERQ-containing proteins identified with the KFERQ finder as CMA substrates. (**A**) Lysosomal degradation of the indicated proteins was measured in NIH 3T3 cells, control (Ctr) or stably KD for LAMP-2A (L2AKD). Cells were treated for 12–24 h with lysosomal inhibitors (Lys inh = NH_4_Cl 20 mM and leupeptin 100 μM), collected and subjected to SDS-PAGE and immunoblot. Proteins were divided into three groups according to the presence (KFERQ-like motif) or absence (no motif) of a KFERQ motif and the dependence on LAMP-2A for their degradation (CMA degradation) or independence of the CMA receptor (lysosomal but not CMA). GAPDH is shown as a control for a known CMA substrate. Graphs show densitometric values of the indicated proteins per group upon normalization to red ponceau staining of the respective membranes. Values are presented as folds over the densitometric intensity in samples nontreated with the lysosomal proteolysis inhibitors. (**B**) Immunoblot for LAMP-2A and LC3 in the same cells as controls for KD and lysosomal inhibitor efficiency, respectively. ACACA, Acetyl-CoA carboxylase 1; ALIX, apoptosis-linked gene 2-interacting protein X; ATGL, adipose triglyceride lipase; BCL2, apoptosis regulator Bcl-2; CDKN2A, cyclin-dependent kinase inhibitor 2; CMA, chaperone-mediated autophagy; Ctr, control; CYLD, Ubiquitin carboxyl-terminal hydrolase CYLD; GAPDH, glyceraldehyde-3-phosphate-dehydrogenase; H2A.X, H2A histone family member; KD, knocked down; LAMP-2A, lysosome-associated membrane protein type 2A; LC3, microtubule-associated protein 1 light chain 3 beta; L2AKD, LAMP-2A knocked down; PSA5, proteasome subunit alpha type-5.
(TIF)

S10 Fig Analysis of proteins containing only N-bearing motifs. **(A)** Percentage of proteins in the human proteome (filtered for reviewed entries) harboring N motifs. (**B**) Distribution of the number of N motifs per protein. Total number of proteins included, 767. (**C**) Enrichment of N-bearing motifs in different subcellular compartments. Compartments of statistically significant difference from the whole proteome (chi-squared, *p < 0.05) are highlighted. Detailed statistics can be found in **S8 Table**. (**D**) Enrichment for a custom selected group of GO terms for biological processes (**S5 Table**) in human proteins containing only one N-bearing motif (total number of proteins, 632). The 10 most enriched terms (by combined score) are displayed. Numbers in the bars are the percentages of motif-containing proteins in the proteins annotated for each term. See **S9 Table** for additional details. GO, gene ontology.
(TIF)

S1 Table Analysis of amino acid solvent exposure in KFERQ-like motifs of experimentally confirmed CMA substrates. CMA, chaperone-mediated autophagy.
(XLSX)

S2 Table Domain localization and PTMs of KFERQ-like motifs in experimentally confirmed substrates. PTM, posttranslational modification.
(XLSX)

S3 Table Proteins selectively conserved in CMA-able species. CMA, chaperone-mediated autophagy.
(XLSX)

S4 Table Evolutionary conservation of CMA components (effectors and modulators of CMA activity). CMA, chaperone-mediated autophagy.
(XLSX)

S5 Table List of GO-terms used for the protein set enrichment analysis. GO, gene ontology.
(XLSX)

S6 Table Chi-squared results for statistics of protein localization. (XLSX)

S7 Table Top five enriched GO terms per motif group, based on the combined score. GO, gene ontology.
(XLSX)

S8 Table Chi-squared results for statistics of localization of N motif–bearing proteins. (XLSX)

S9 Table Top 10 enriched GO terms in proteins containing one single N-bearing motif. GO, gene ontology.
(XLSX)

## Abbreviations

AKT1: RAC-alpha serine/threonine-protein kinase
BLAST: Basic Local Alignment Search Tool
Cath A: protective protein/cathepsin A
CHK1: checkpoint kinase 1
CMA: chaperone-mediated autophagy
eF1a: Elongation factor 1-alpha
eMI: endosomal microautophagy
GAPDH: glyceraldehyde-3-phosphate dehydrogenase
GFAP: Glial fibrillary acidic protein
GO: gene ontology
HIF1a: hypoxia-inducible factor 1-alpha
HSC70: heat shock cognate 71 kDa protein
HSP40: DnaJ homolog subfamily B member 1
HSP90: heat shock protein HSP 90
KD: knocked down
LAMP-2A: lysosome-associated membrane protein type 2A
LC3-II: microtubule-associated proteins 1A/1B light chain 3B
NFAT: nuclear factor of activated T cells
NRF-2: nuclear factor erythroid 2-related factor 2
PARK7: Parkinsonism associated deglycase
PAT: perilipin/ADRP/TIP47
PHLPP1: PH domain leucine-rich repeat-containing protein phosphatase 1
PLIN3: perilipin 3
PTM: posttranslational modification
Rab11: Ras-related protein Rab-11A
RAC1: Ras-related C3 botulinum toxin substrate 1
RARα: Retinoic acid receptor alpha
SLiM: small linear motif
