# Correction: Proteome-wide analysis of chaperone-mediated autophagy targeting motifs

**DOI:** 10.1371/journal.pbio.3001550

**Published:** 2022-02-04

**Authors:** Philipp Kirchner, Mathieu Bourdenx, Julio Madrigal-Matute, Simoni Tiano, Antonio Diaz, Boris A. Bartholdy, Britta Will, Ana Maria Cuervo

In the Abstract, Results, Discussion, and legend for S8 Fig, the hyperlink to the “KFERQ finder” tool is inactive. Please see the correct link here: https://rshine.einsteinmed.edu/.
