# Supplementary figures and images for: Proteome-wide analysis of chaperone-mediated autophagy targeting motifs
Source: PLoS Biol. 2019 May 31;17(5):e3000301. doi: 10.1371/journal.pbio.3000301 (PMC6561683; doi:10.1371/journal.pbio.3000301)

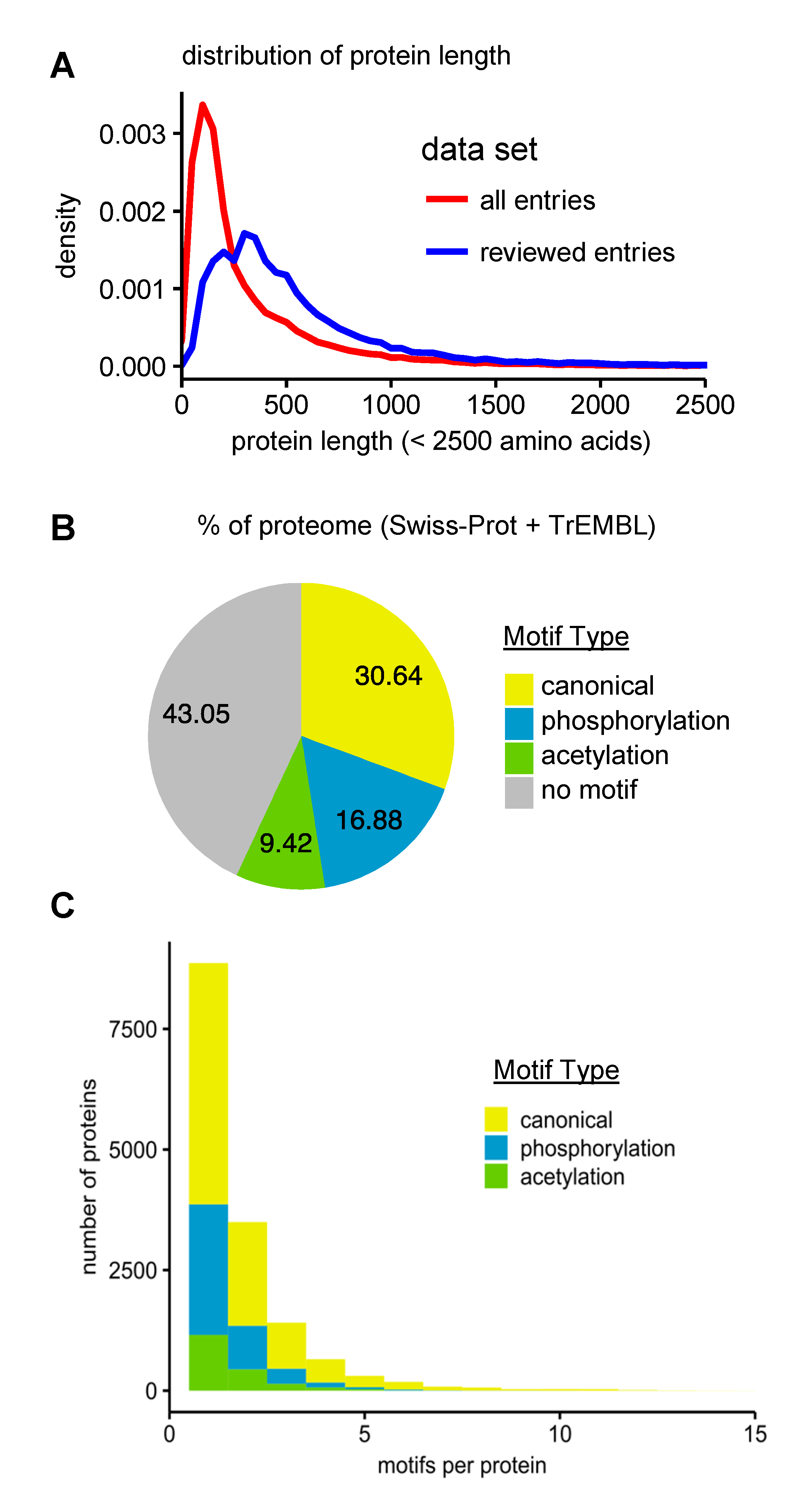

Supplement: S1 Fig — (A) Comparison between the length of proteins in the reviewed Swiss-Prot and complete UniProt human proteome. Swiss-Prot entries were filtered from the human proteome by their revision status in the UniProtKB. The density of proteins with a length <2,500 amino acids is shown. (B) Percentage of proteins with the different types of KFERQ-like motifs in the unfiltered human proteome. The data are grouped and displayed as in Fig 1B. (C) Distribution of the number of motifs per protein. For each protein, the number of motifs following the hierarchical priority described in Fig 1B was calculated. The bars in the histogram are colored according to the motif types. (TIF) [file pbio.3000301.s001.tif]

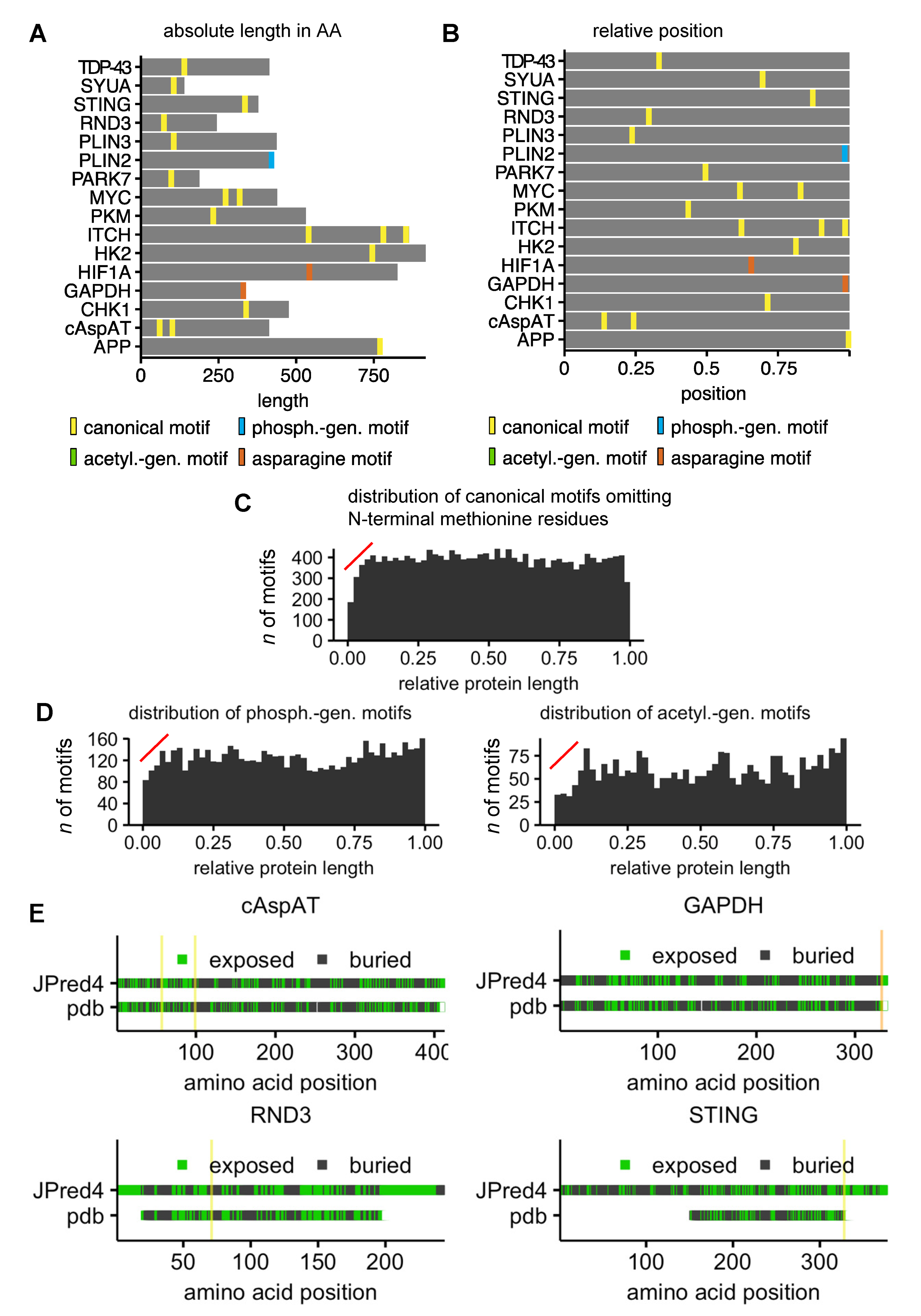

Supplement: S2 Fig — Absolute (A) and relative (B) positions of the KFERQ-like motifs in experimentally validated CMA substrates (taken from published literature summarized in [4]). The position and type of motif are indicated by colored boxes (yellow, canonical; blue, phosphorylation-generated; green, acetylation-generated). Red boxes indicate experimentally validated canonical motifs in which an N is found in place of a Q (these motifs are not included in the proteome-wide analysis because, in contrast to the other motifs, they require additional unknown circumstances to target a protein towards CMA). (C) Histogram of the frequency of canonical motifs along the protein length excluding initiator methionine residues. The data are presented as in Fig 2A. Red line indicates the slope of the reduction in KFERQ-like motifs. (D) Distribution of phosphorylation- or acetylation-generated motifs along the protein length. The length of the proteins is normalized to a scale from 0 (N-terminus) to 1 (C-terminus). The histogram shows the count of motifs at the relative position with a bin size of 0.02. (E) Examples of protein secondary structure analyses in validated CMA substrates. The relative solvent exposure of amino acids was calculated from pdb crystal structures or predicted using JPred4. Amino acids with a relative solvent exposure below 25% were considered buried (note that for RND3 and STING, pdb data were only available for a fragment of the protein, shown here aligned with the full sequence). The vertical yellow lines indicate the positions of the KFERQ-like motifs (the central amino acid of a motif marks the motif position). CMA, chaperone-mediated autophagy. (TIF) [file pbio.3000301.s002.tif]

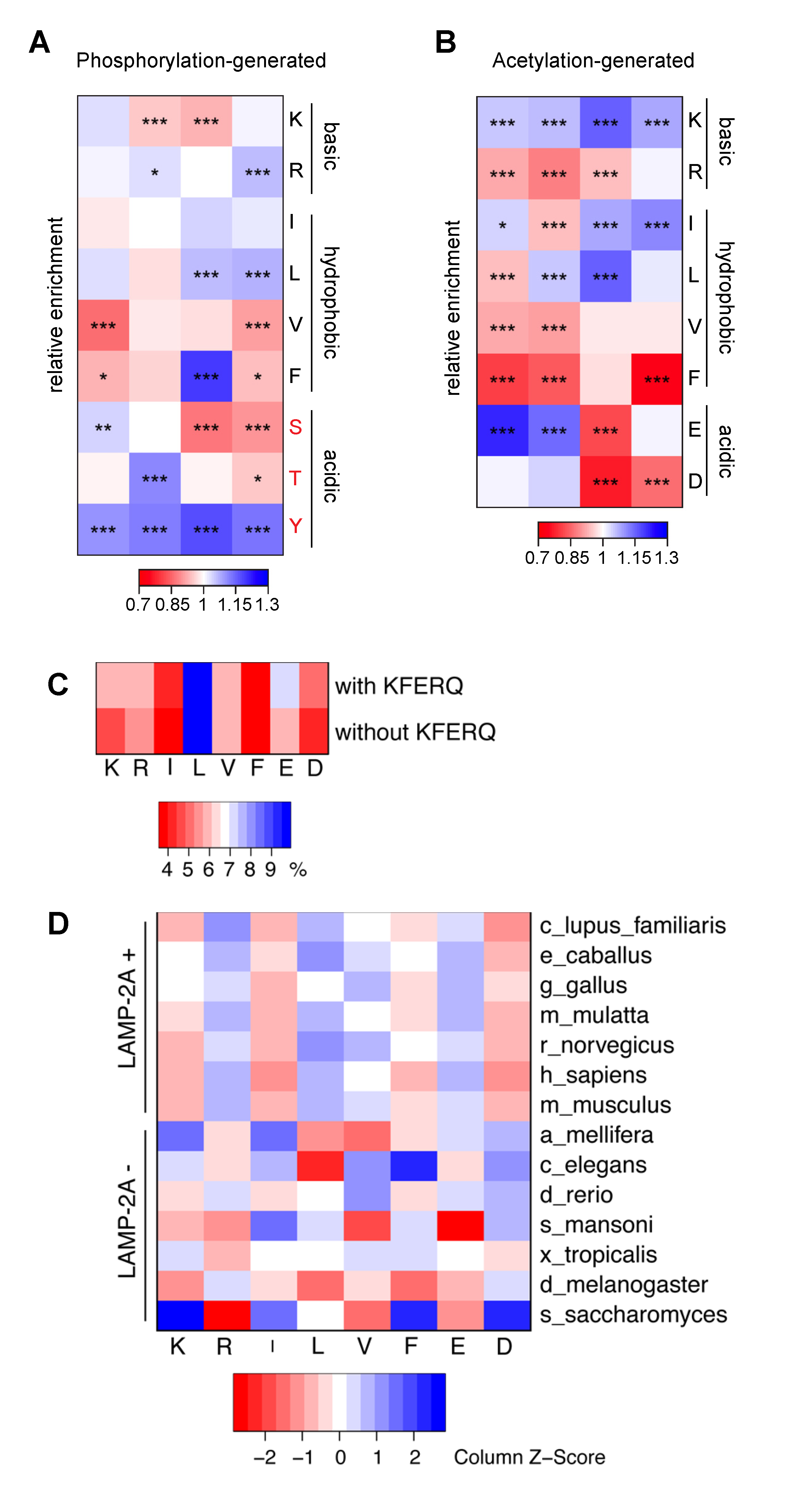

Supplement: S3 Fig — Comparison of amino acid frequencies at each position in phosphorylation-generated (A) and acetylation-generated (B) motifs from the human proteome and from a permutated proteome. Amino acid counts from Fig 3B and 3C were divided by the counts in motifs from permutated proteins. To superimpose motifs starting or ending with a glutamine, motifs starting with a glutamine are mirrored. The amino acid positions are given, relative to the glutamine (−1 = closest and −4 = furthest away). Means are from 40 random samples of 10% of the data sets each. ***p < 0.001, **p < 0.01, *p < 0.5. The p-values from two-sided t tests are corrected (Bonferroni) by the number of comparisons (n = 32). (C) Frequency of total amino acids in proteins containing KFERQ-like motifs and proteins without a motif. For each protein in the unfiltered human data set, the percentage of amino acids that can become part of a KFERQ-like motif was calculated. The data set was then split into the pool of proteins with and without KFERQ-like motifs. The heat map displays the amino acid percentages in each group. (D) Amino acid frequencies calculated as in (C) but over the whole proteomes of species with (LAMP-2A+ = able to perform CMA) and without (LAMP-2A− = unable to perform CMA) the CMA receptor LAMP-2A. The analysis for presence of LAMP-2A in different species is presented in detail in S4C Fig. Amino acid percentages are scaled to standard normal distributions over the heat map columns to normalize differences in the relative abundance of individual amino acids. CMA, chaperone-mediated autophagy; LAMP-2A, lysosome-associated membrane protein type 2A. (TIF) [file pbio.3000301.s003.tif]

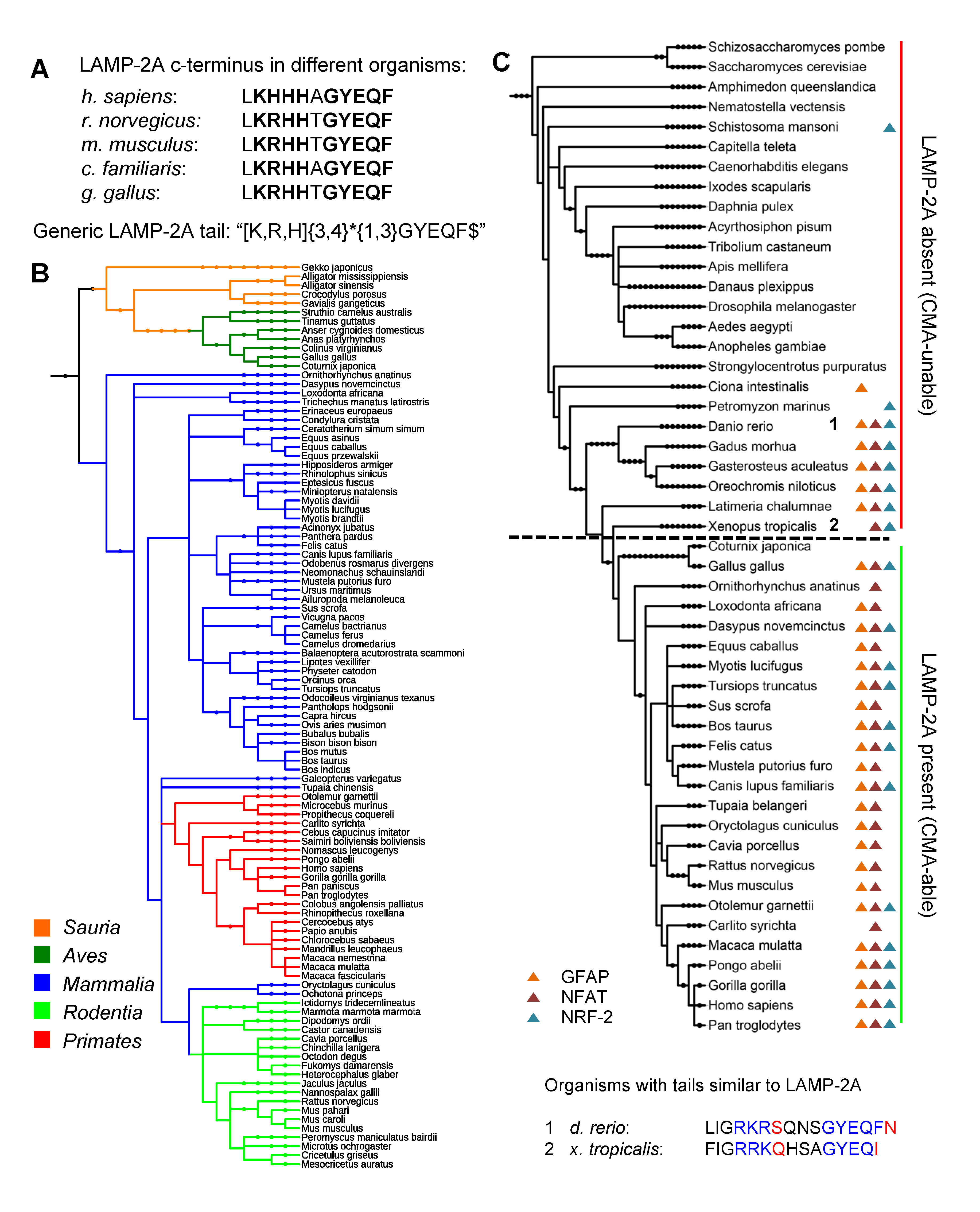

Supplement: S4 Fig — (A) C-termini of LAMP-2A isoforms in species with experimentally demonstrated CMA activity and regular expression for identification of LAMP-2A homologues. (B) Species with a homologue to human LAMP-2A identified by a BLAST search against the C-terminal (100 amino acids) region and further filtered for exact matches to the pattern of the human LAMP-2A C-terminus. If multiple hits are returned, the one closest in length to human LAMP-2A is chosen. The evolutionary relation between all species with LAMP-2A homologues is shown as an evolutionary tree (visualization from itol.embl.com). Sub-trees for interesting nodes (Sauria, Aves, Mammalia, Rodentia, and Primates) are color coded. (C) Set of species used in the analysis of the evolutionary conservation of motifs. Organisms with available protein sequence information are selected from the TreeFam species tree (March 2013, treefam.org). In rare cases in which few EggNOG orthologs were found for a particular species from the treefam.org database, this conflict was manually resolved by selecting a different subspecies of this species with higher coverage in the EggNOG database, when available. The list of species is combined with the analysis of LAMP-2A to construct a set of 50 species classified by the presence or absence of a LAMP-2A homologue. Species with unclear status (e.g., containing imperfectly matching LAMP-2A tails) are omitted to simplify the classification. Upon analysis of the conservation of other components of the CMA machinery (Fig 4C), three proteins, GFAP, NFAT, and NRF2, showed only partial conservation but were selectively enriched in CMA-able species. The presence of these proteins in a species is indicated through color coded triangles. BLAST, Basic Local Alignment Search Tool; CMA, chaperone-mediated autophagy; GFAP, Glial fibrillary acidic protein; LAMP-2A, lysosome-associated membrane protein type 2A; NFAT, nuclear factor of activated T cells; NRF2, nuclear factor erythroid 2-related factor 2. (T [file pbio.3000301.s004.tif]

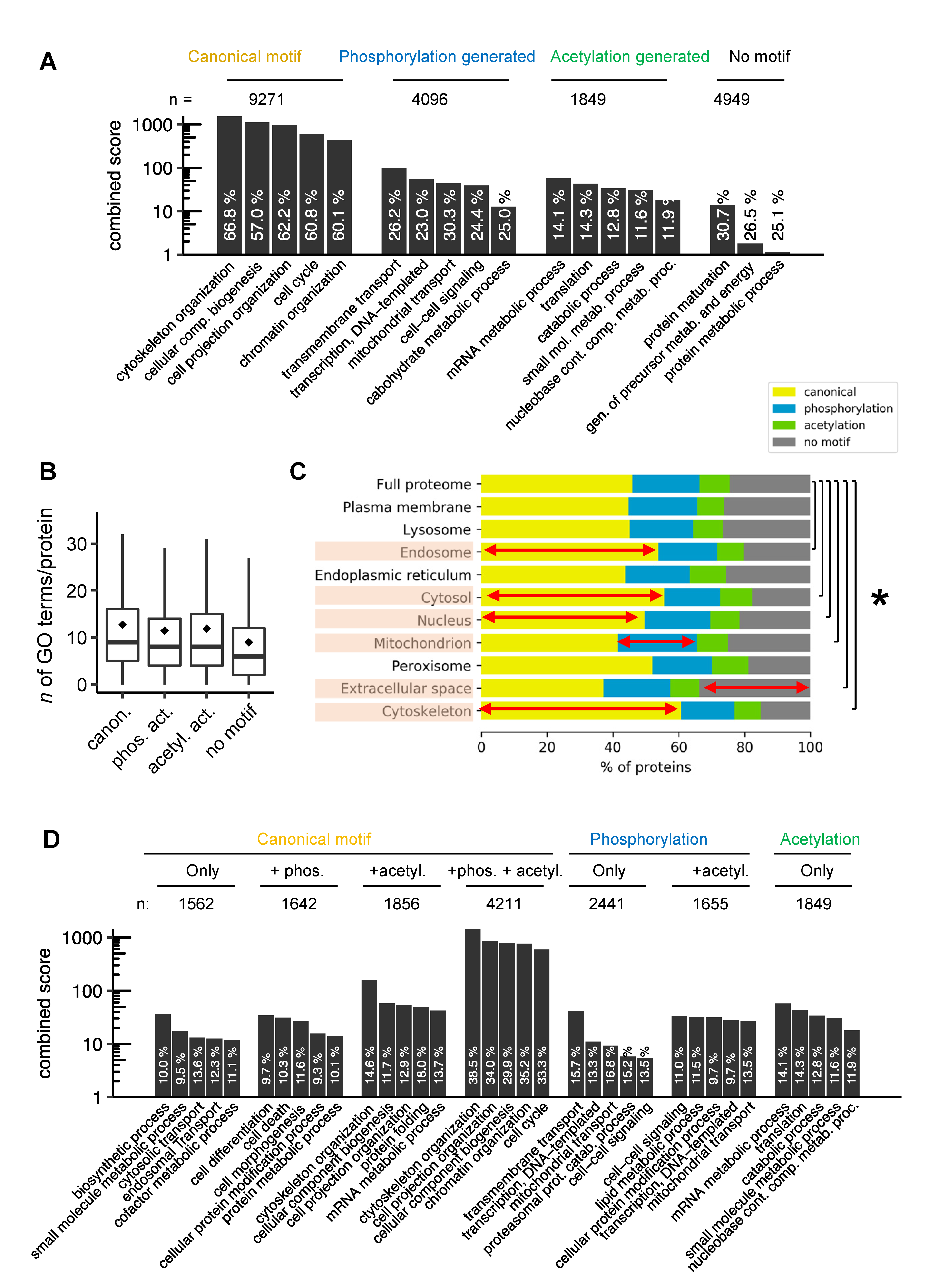

Supplement: S5 Fig — (A) Enrichment for a custom selected group of GO terms for biological processes (S5 Table) in human proteins, grouped by motif type (as in Fig 1B) or with no motif. For each group, the five most enriched terms (by combined score = −loge(p-value)*z-score) are displayed. Numbers on top indicate the total number of proteins in each group and numbers in the bars the percentage of motif-containing proteins in the proteins annotated for each term. See S7 Table for additional details. (B) Number of GO annotations per protein in the groups analyzed in Fig 5A. For each protein, the number of GO terms annotated in the UniProt database is calculated. The box plots show median, 25th, and 75th percentiles. Whiskers are 1.5 * IQR, and diamonds show the data set mean. Outliers are omitted for clarity. (C) Enrichment of KFERQ-like motifs in different compartments. A highlighted compartment indicates statistically significant difference from the whole proteome (chi-squared, *p < 0.05). Detailed statistics can be found in S6 Table. Red arrows show the most enlarged compartment. (D) Enrichment for a custom selected group of GO terms for biological processes (S5 Table) in human proteins grouped by combinations of canonical, phosphorylation-, or acetylation-generated motifs. For each group, the five most enriched terms are displayed. See S7 Table for additional details. GO, gene ontology. (TIF) [file pbio.3000301.s005.tif]

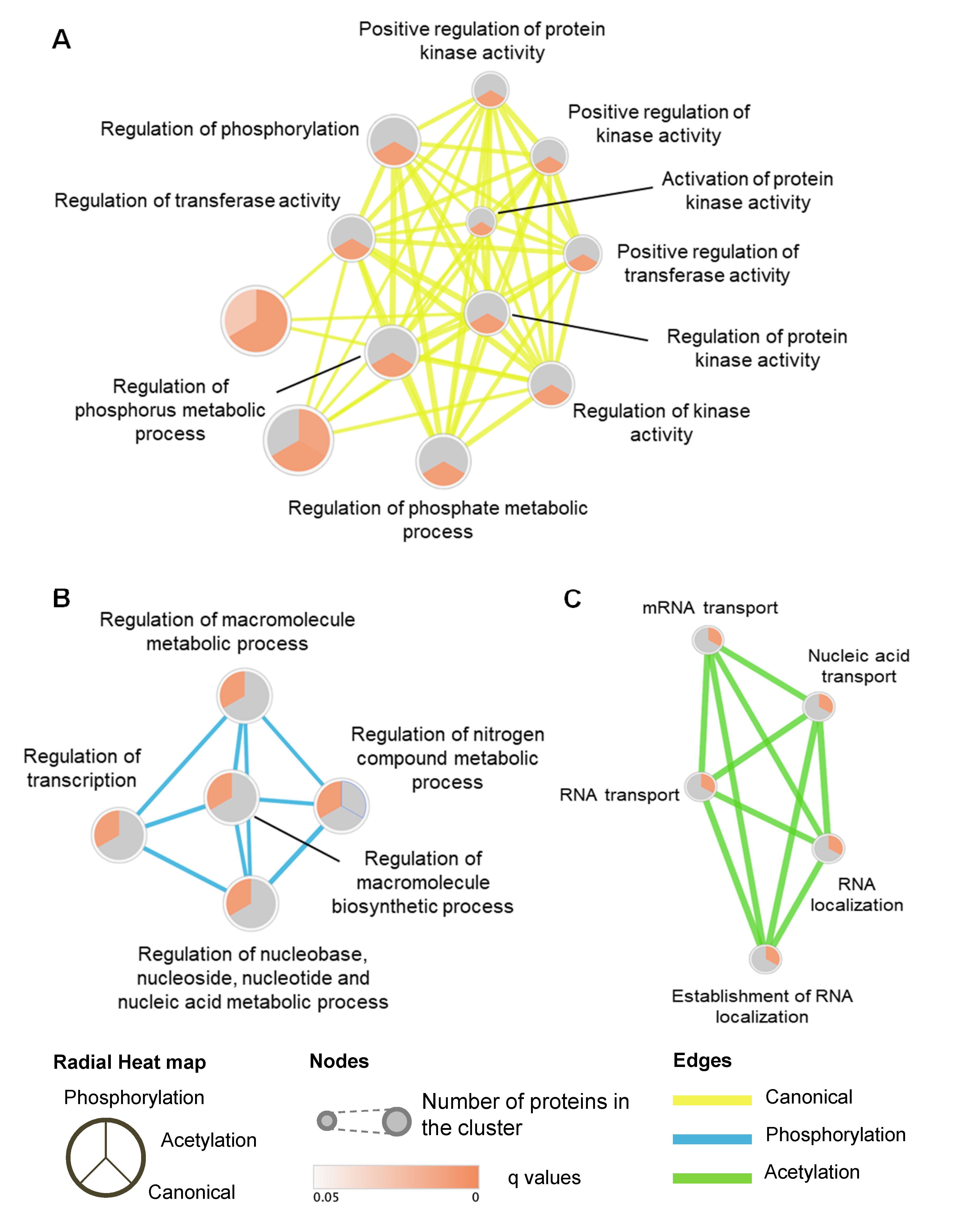

Supplement: S6 Fig — (A-C) Clusters of GO terms associated with specific types of motifs: (A) canonical, (B) phosphorylation-generated, and (C) acetylation-generated. The nodes are radial heat maps in which the size is proportional to the number of proteins within the given annotation and the color intensity of the filling depicts association with a specific kind of motif (distribution indicated in the bottom of the figure). Edges represent similarity between nodes. GO, gene ontology. (TIF) [file pbio.3000301.s006.tif]

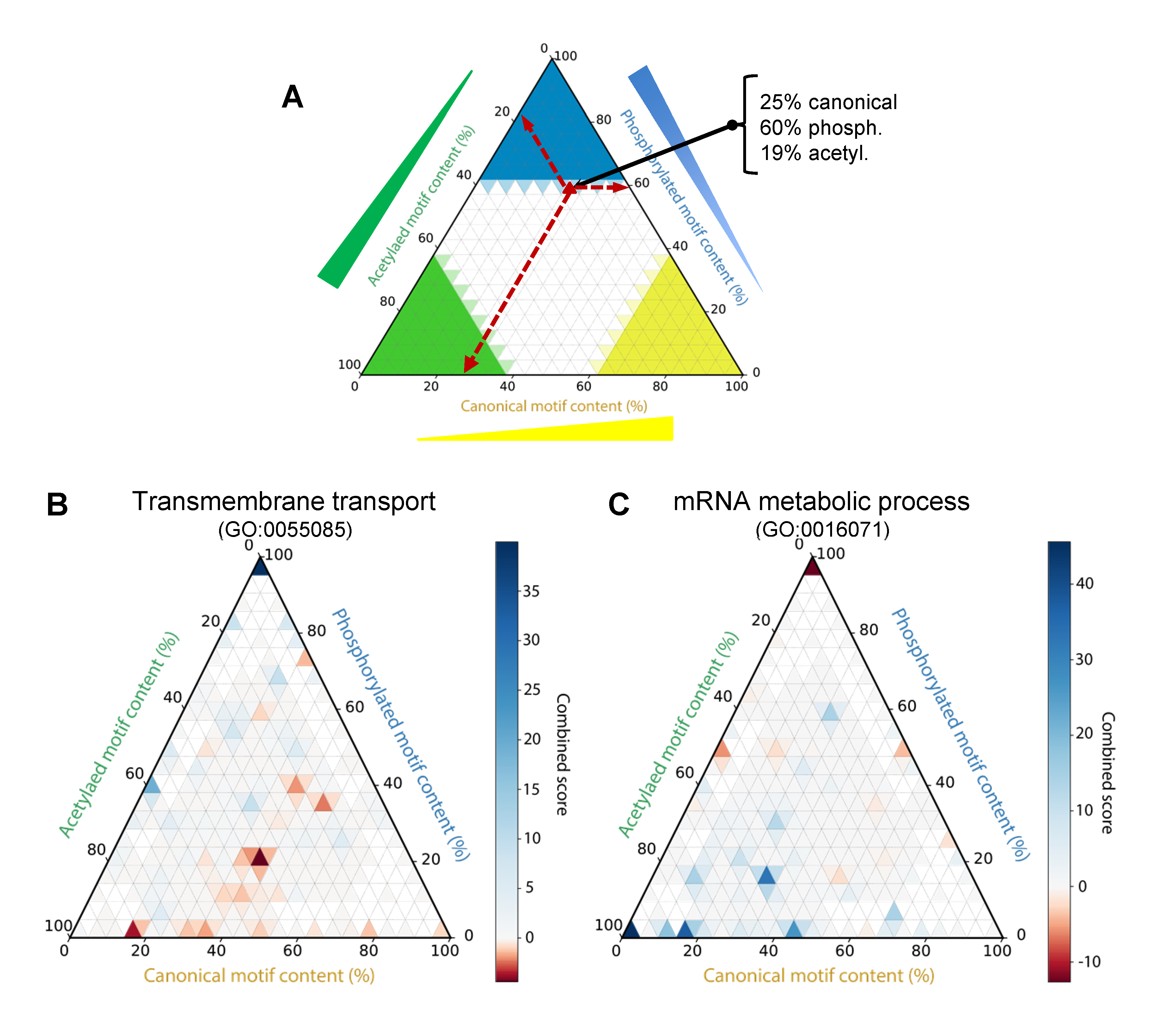

Supplement: S7 Fig — (A) Cartoon explaining the triangle plots used to display protein groups characterized by fractional motif content. For each protein, the fraction (0 to 100 percent) of canonical, phosphorylation-, and acetylation-generated motifs is calculated. Each side of the triangle plot represents the fraction of one motif class in 5% steps. The smaller triangles in the plot are bins of proteins with a specific motif combination. For example, proteins with a high content of canonical motifs will appear in the lower right corner (yellow), with proteins containing exclusively canonical motifs at the extreme right. The same is shown for phosphorylation-generated (blue) and acetylation-generated (green) motifs. Locations of hypothetical examples of motif composition percentages are shown with red lines. (B, C) Examples of GO terms showing enrichment for phosphorylation-generated (B) and acetylation-generated (C) motifs. GO, gene ontology. (TIF) [file pbio.3000301.s007.tif]

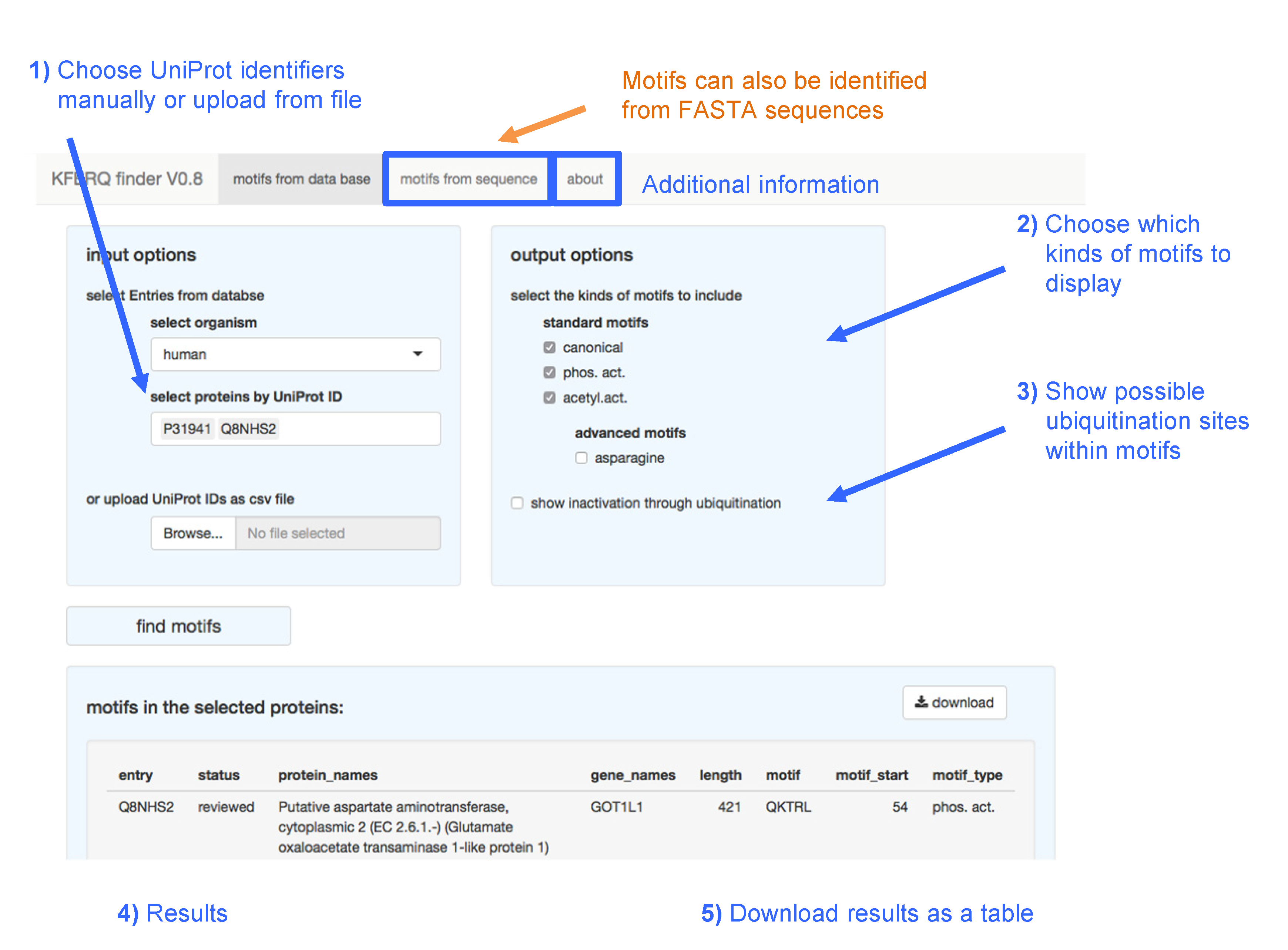

Supplement: S8 Fig — Screenshot of the free online software “KFERQ finder” for analysis of KFERQ-like motifs in protein sequences. Steps and options for database analysis are shown. (TIF) [file pbio.3000301.s008.tif]

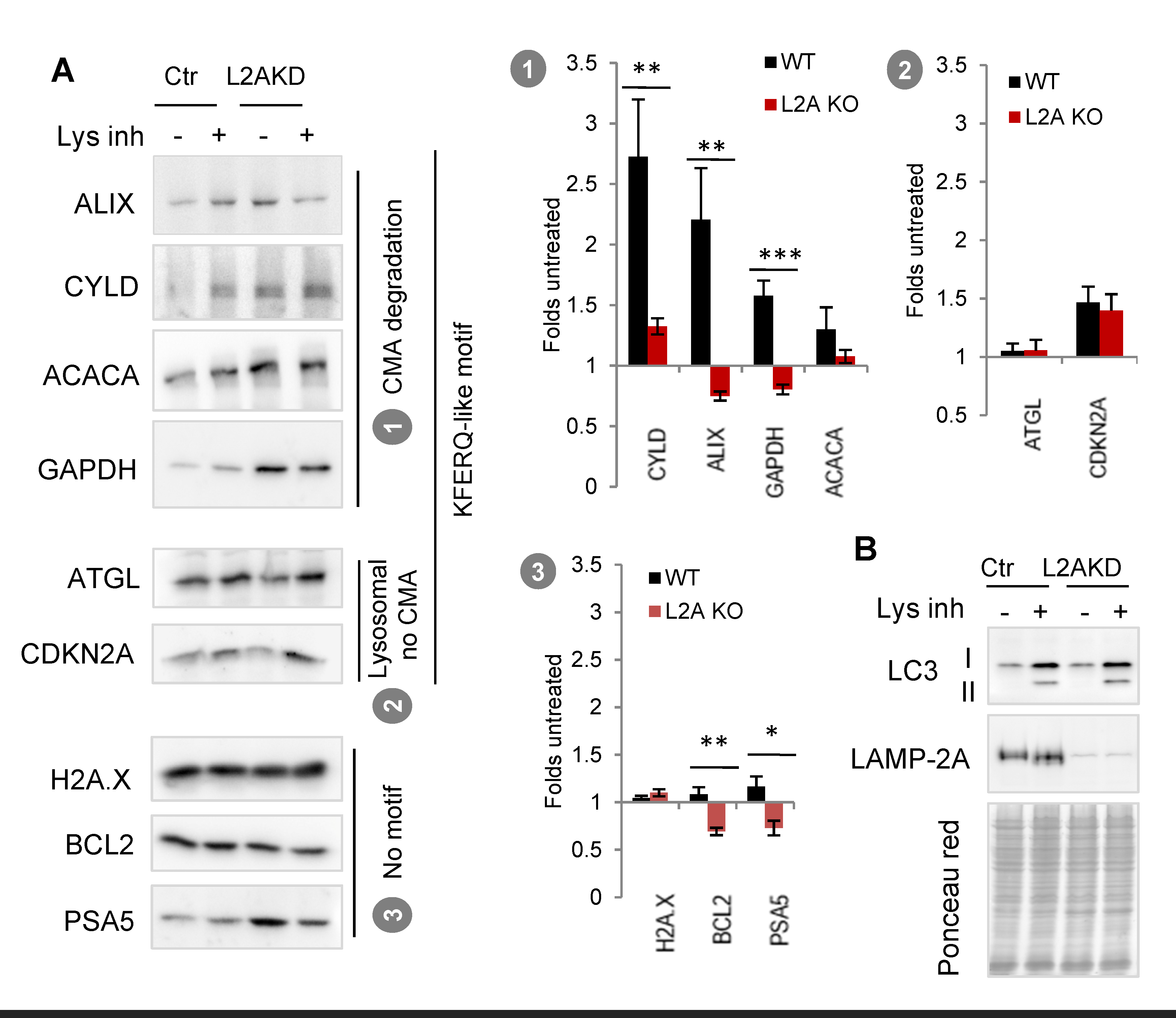

Supplement: S9 Fig — (A) Lysosomal degradation of the indicated proteins was measured in NIH 3T3 cells, control (Ctr) or stably KD for LAMP-2A (L2AKD). Cells were treated for 12–24 h with lysosomal inhibitors (Lys inh = NH4Cl 20 mM and leupeptin 100 μM), collected and subjected to SDS-PAGE and immunoblot. Proteins were divided into three groups according to the presence (KFERQ-like motif) or absence (no motif) of a KFERQ motif and the dependence on LAMP-2A for their degradation (CMA degradation) or independence of the CMA receptor (lysosomal but not CMA). GAPDH is shown as a control for a known CMA substrate. Graphs show densitometric values of the indicated proteins per group upon normalization to red ponceau staining of the respective membranes. Values are presented as folds over the densitometric intensity in samples nontreated with the lysosomal proteolysis inhibitors. (B) Immunoblot for LAMP-2A and LC3 in the same cells as controls for KD and lysosomal inhibitor efficiency, respectively. ACACA, Acetyl-CoA carboxylase 1; ALIX, apoptosis-linked gene 2-interacting protein X; ATGL, adipose triglyceride lipase; BCL2, apoptosis regulator Bcl-2; CDKN2A, cyclin-dependent kinase inhibitor 2; CMA, chaperone-mediated autophagy; Ctr, control; CYLD, Ubiquitin carboxyl-terminal hydrolase CYLD; GAPDH, glyceraldehyde-3-phosphate-dehydrogenase; H2A.X, H2A histone family member; KD, knocked down; LAMP-2A, lysosome-associated membrane protein type 2A; LC3, microtubule-associated protein 1 light chain 3 beta; L2AKD, LAMP-2A knocked down; PSA5, proteasome subunit alpha type-5. (TIF) [file pbio.3000301.s009.tif]

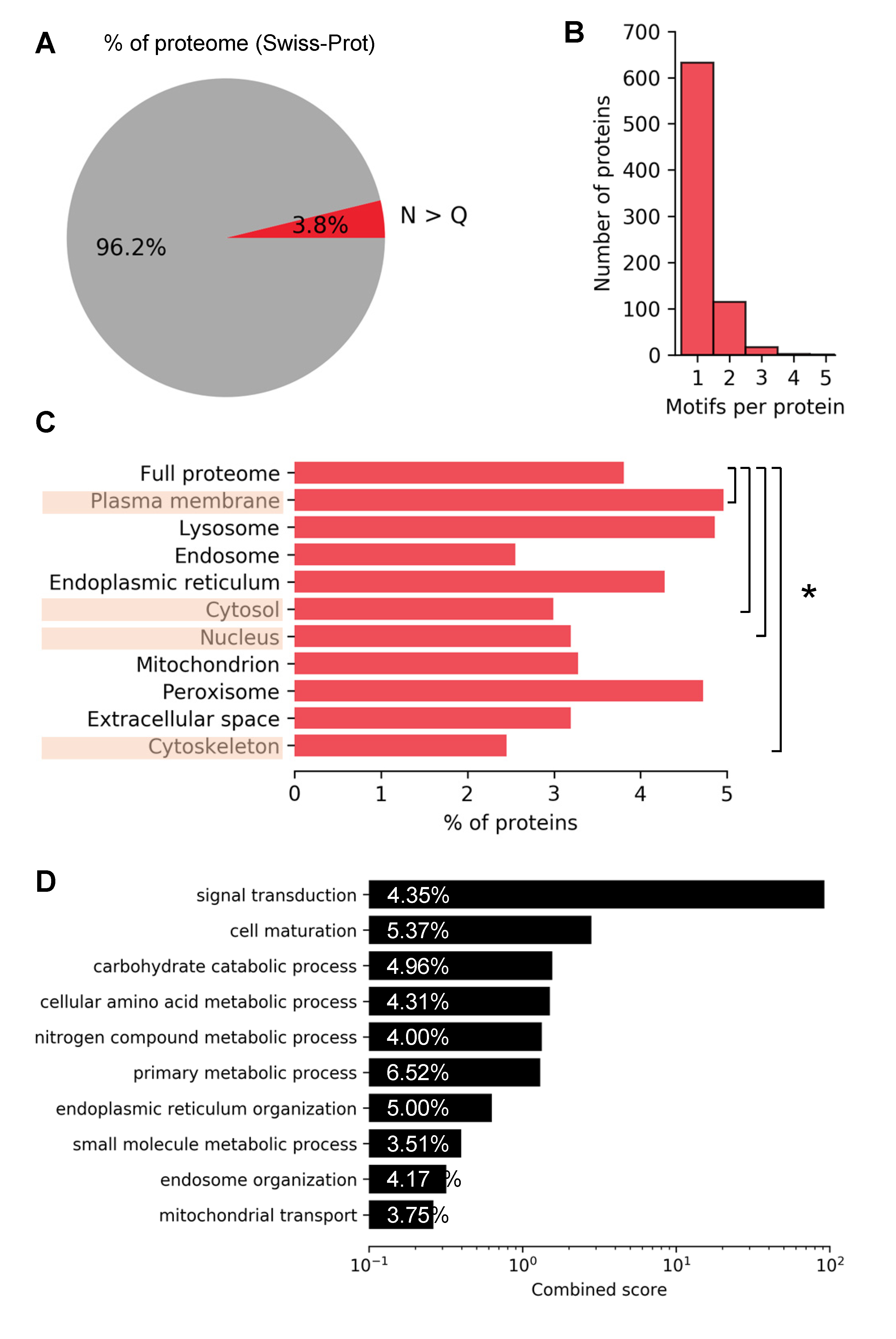

Supplement: S10 Fig — (A) Percentage of proteins in the human proteome (filtered for reviewed entries) harboring N motifs. (B) Distribution of the number of N motifs per protein. Total number of proteins included, 767. (C) Enrichment of N-bearing motifs in different subcellular compartments. Compartments of statistically significant difference from the whole proteome (chi-squared, *p < 0.05) are highlighted. Detailed statistics can be found in S8 Table. (D) Enrichment for a custom selected group of GO terms for biological processes (S5 Table) in human proteins containing only one N-bearing motif (total number of proteins, 632). The 10 most enriched terms (by combined score) are displayed. Numbers in the bars are the percentages of motif-containing proteins in the proteins annotated for each term. See S9 Table for additional details. GO, gene ontology. (TIF) [file pbio.3000301.s010.tif]
